# Zhenyuan Solid Drink Ameliorates Inflammation and Oxidative Stress in Isoproterenol‐Induced Ischemic Myocardial Infarction via TLR4/NF‐κB Pathway and PI3K/AKT1/NRF2 Pathway in Rats

**DOI:** 10.1002/fsn3.71798

**Published:** 2026-04-19

**Authors:** Jiawen Huang, Tian Yu, Guoyi Li, Kechao Nie, Donghua Liu, Cong Meng, Xiaoling Shen, Linchun Fu, Liang Long, Zhuotao Fu, Zhitong Deng, Yingjie Hu

**Affiliations:** ^1^ Science and Technology Innovation Center, Guangzhou University of Chinese Medicine Guangzhou China; ^2^ Department of Integrated Traditional Chinese & Western Medicine The Second Xiangya Hospital, Central South University Changsha China; ^3^ First Affiliated Hospital of Guangzhou University of Chinese Medicine Guangzhou China; ^4^ The Affiliated Traditional Chinese Medicine Hospital, Guangzhou Medical University Guangzhou China; ^5^ Haikou Hospital of Traditional Chinese Medicine Haikou China

**Keywords:** chinese medicine, diet therapy, inflammation, ischemic myocardial infarction, MD simulation, oxidative stress

## Abstract

Zhenyuan Solid Drink (ZYSD) is a formulation consisting of 6 medicinal herbs that can be used as both food and medicinal materials in China. This comprehensive analysis aims to illuminate the potential therapeutic effects and underlying mechanisms of ZYSD in the context of ischemic myocardial infarction. Marker components in ZYSD were identified by high performance liquid chromatography (HPLC) through comparison with reference compounds. Isoproterenol (ISO)‐induced rats were employed as the in vivo model of ischemic myocardial infarction. Subsequently, a multi‐tiered approach integrating cardiac color Doppler ultrasound, cardiac enzyme marker analysis, inflammatory factor detection, gut microbiota profiling, network pharmacology, molecular docking, molecular dynamics (MD) simulation, and western blot analysis was employed to elucidate the effects and underlying mechanisms of ZYSD, with intervention in alleviating metoprolol tartrate as positive control. Ten compounds, derived from 6 medicinal plants, in ZYSD were identified as marker components for the quality control via HPLC. Animal studies confirmed the preventive and therapeutic efficacy of ZYSD against ischemic myocardial infarction, as evidenced by the improvement of cardiac function and the attenuation of inflammation and oxidative stress. Using network pharmacology and molecular docking, the active constituents including marker compounds of ZYSD exhibited strong binding affinity to their key targets, including AKT1 and NRF2. MD simulation further indicated the binding interaction between ZYSD components and AKT1. Western blot analysis results further verified that ZYSD regulates the TLR4/NF‐κB pathway and PI3K/AKT1/NRF2 pathway. Collectively, our findings demonstrate that ZYSD mitigates inflammation and oxidative stress, potentially through the TLR4/NF‐κB and PI3K/AKT1/NRF2 pathways, thereby providing a novel strategy for the prevention and treatment of ischemic myocardial infarction.

## Graphical abstract

ZYSD exerts curative and preventive cardioprotection against isoproterenol‐induced myocardial infarction through synergistic interaction with probiotics, exerting antioxidant and anti‐inflammatory effects.

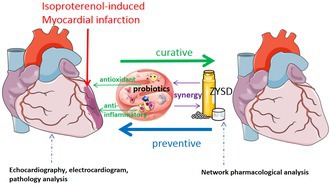

**Abbreviations**
CK‐MB: creatine kinase‐MB isoenzyme
ECG: electrocardiogram
EDV: end‐diastolic volume
ES: *Epimedium sagittatum* (Sieb. Et Zucc.) Maxim
ESV: end‐systolic volume
GSH: glutathione
GU: *Glycyrrhiza uralensis* Fisch
HAART: highly active antiretroviral therapy
H&E: hematoxylin and eosin
HPLC: high performance liquid chromatography
LOOH: lipid hydroperoxides
LVEDD: end‐diastolic left ventricular internal diameter
LVESD: end‐systolic left ventricular internal diameter
MM/GBSA: molecular mechanics/generalized born surface area
NPT: isothermal‐isobaric ensemble
NVT: canonical ensemble
PA: *Panax ginseng* C. A. Mey
PC: *Poria cocos* (Schw.) Wolf
SM:
*Salvia miltiorrhiza* Bge
SOD: superoxide dismutase
TBARS: thiobarbituric acid reactive substances
TCM: Traditional Chinese Medicine
ZO:
*Zingiber officinale* Rosc
ZYSD: Zhenyuan Solid Drink

## 1 Introduction

Ischemic myocardial infarction represents a leading cause of mortality worldwide, resulting from impaired coronary blood flow and myocardial oxygen deprivation, which ultimately triggers severe myocardial injury (Vaduganathan et al. 2022). The pathogenesis of this disorder is closely linked to oxidative stress and inflammatory responses, which initiate a cascade of biochemical and molecular alterations that subsequently give rise to profound functional and morphological changes in the myocardium (Nagoor Meeran et al. 2019). A range of diagnostic modalities including electrocardiography, serum cardiac enzyme levels, echocardiography, and histopathological examination allow for the direct or indirect identification of myocardial infarction.

Isoproterenol, a β‐adrenergic receptor agonist, has been widely reported to induce myocardial infarction‐like necrosis in rat myocardium through oxidative stress pathways, which mimics the human pathological changes seen in ischemic necrosis secondary to vascular occlusion (Xu et al. 2011). Metoprolol tartrate, a β1‐selective adrenergic receptor blocker, is commonly used in the management of ischemic myocardial infarction by reducing heart rate, lowering myocardial oxygen demand, and inhibiting adverse remodeling. Despite potential adverse effects such as gastrointestinal disturbances, its clinical value in prevention and treatment of ischemic myocardial infarction is well recognized, and existing evidence supports its beneficial role in improving patient outcomes (Podlesnikar et al. 2020). Metoprolol tartrate was used as a positive control in the present study.

The formulation of ZYSD includes *Epimedium sagittatum* (Sieb. Et Zucc.) Maxim, *Poria cocos* (Schw.) Wolf, *Panax ginseng* C. A. Mey., *Salvia miltiorrhiza* Bge., *Zingiber officinale* Rosc., and *Glycyrrhiza uralensis* Fisch. All of these ingredients are recognized as safe for consumption in the Catalog of Both Medicinal and Edible Substances of the People's Republic of China (China TMoHo 2002). ZYSD is a food product prepared from the water extract of the above 6 herbs, which makes it suitable for long‐term healthcare use. Previous studies have verified that the bioactive components of ZYSD exert favorable effects against cardiovascular diseases. Icariin alleviates diabetic cardiomyopathy by activating Nrf2‐dependent antioxidant and mitochondrial pathways (Zhang et al. 2026); Poria cocos improves cardiac function in rats with chronic heart failure via inhibiting renal aquaporin‐2 expression (Wu et al. 2014); Panax ginseng and its active ingredients produce remarkable cardioprotection by ameliorating myocardial energy metabolism remodeling (Zhou et al. 2024); and extracts of *Salvia miltiorrhiza* as well as its active components including salvianolic acid A and tanshinone IIA also exhibit protective effects against cardiovascular diseases (Guo et al. 2020; Dawuti et al. 2023; Wu et al. 2024). Collectively, these pieces of evidence indicate that ZYSD may possess therapeutic potential for the intervention of ischemic myocardial infarction.

Given the high global burden of ischemic myocardial infarction and the clinical demand for safer and more effective intervention strategies, the exploration of cardioprotective agents derived from medicinal and edible homologous herbs has become particularly necessary and urgent. This study initially evaluated the potential of ZYSD in the treatment of ischemic myocardial infarction by using network pharmacology to predict its intervention targets. Subsequent animal experiments were designed to confirm the preventive and therapeutic effects of ZYSD on ischemic myocardial infarction and to elucidate its mechanisms of action, with comparative analysis against metoprolol tartrate. This research lays a foundation for the development of ZYSD as a specialized medical food for ischemic myocardial infarction and provides a novel approach to cardiovascular health protection.

## 2 Methods and Materials

### 2.1 Drugs and Reagents

ZYSD used in this study was a dried water extract prepared from a mixture of TCM decoction pieces of *Epimedium sagittatum*, *Zingiberis Rhizoma*, *Glycyrrhiza uralensis*, *Panax ginseng*, *Poria cocos*, and *Salvia miltiorrhiza* , at a mass ratio of 6:3:3:2:2:2, respectively. The TCM decoction pieces were provided by the Yang Ming Company in Kunming, Yunnan Province, China. Their botanical identities were authenticated by the corresponding author (Y.H.) as *Epimedium sagittatum* (Sieb. Et Zucc.) Maxim. (ES), *Zingiber officinale* Rosc. (ZO), *Glycyrrhiza uralensis* Fisch. (GU), *Panax ginseng* C. A. Mey. (PA), *Poria cocos* (Schw.) Wolf (PC) and *Salvia miltiorrhiza* Bge (SM), respectively. Experimental examination confirmed that the quality control indicators of the TCM decoction pieces complied with the requirement of the Chinese Pharmacopeia (2020 Edition). The origins and lot numbers of the TCM decoction pieces are detailed in the accompanying Supplementary Table 1 for reference.

Reference compounds (Lot number), including icariin (110737‐202017), liquiritin (111610‐202209), quercetin (100081‐200907), tanshinone IIA (110766‐202323), and ammonium glycyrrhizinate (110731‐202122) were procured from the China Institute for Drug Control, National Medical Products Administration (NMPA). Epimedin A (E32451), epimedin B (E19891), and epimedin C (E67881) were sourced from Shanghai Jizhi Biochemical Technology Co. Ltd. Salvianolic acid C (C26H20010) and ginsenoside Rb3 (C53H90022) were acquired from Nanjing Chunqiu Biotechnology Co. Ltd. All aforementioned reference substances were of analytical purity. Chromatography‐grade acetonitrile (100108) was obtained from Tianjin Kangkede Technology Co. Ltd.

For a total of 36.0 kg of the mixed TCM decoction pieces prepared in the aforementioned specific mass ratio, pure water of 15 times the amount of decoction pieces was added and boiled by reflux for 3 times, each time 2 h. The extraction solutions were combined and filtered. The combined filtrate was concentrated and vacuum‐dried, powdered to give ZYSD dry powder (Supplementary Figure 1), with an extraction yield rate of 17.0%.

### 2.2 ZYSD Fingerprint Spectrum Determined by HPLC

0.2 g of ZYSD was accurately weighed and placed into a conical flask. Subsequently, 20 mL of 50% ethanol solution was added, after which the weight was recorded. The mixture was then subjected to ultrasonication at 480 watts and 40 kHz for 1 h. After cooling to room temperature, the mixture was shaken thoroughly and centrifuged at 11,000 rpm for 10 min. The supernatant was filtered through a 0.22‐μm microporous membrane to obtain the test solution with a concentration of 10 mg/mL.

The reference substance was dissolved in methanol to prepare stock solutions with the following final concentration of 0.40 mg/mL for liquiritin, 0.10 mg/mL for ginsenoside Rb3, epimedin A for 0.10 mg/mL, epimedin B for 0.10 mg/mL, salvianolic acid C for 0.40 mg/mL, epimedin C for 0.30 mg/mL, icariin for 0.40 mg/mL, quercetin for 0.08 mg/mL, ammonium glycyrrhizinate for 0.75 mg/mL, tanshinone IIA for 0.65 mg/mL, respectively.

Chromatographic analysis was conducted using a Kromasil 100‐5‐C18 (250 mm × 4.6 mm, 5 μm) column. Acetonitrile served as mobile phase A, and water as mobile phase B. The detection wavelength was set at 280 nm, and the column temperature was maintained at 30°C. The flow rate was set at 1 mL/min, and the sample injection volume was 5 μL. The elution gradient is detailed in Supplementary Table 2.

### 2.3 Animal Experiments

A total of 90 male Sprague–Dawley rats, each aged 8 weeks, was sourced from Guangdong Sijia Jingda Biotechnology Co. Ltd. (Quality Certificate Number 430727240100347148, Laboratory Animal Production License: SCXK [Xiang] 2019‐0004). The animals were maintained on a 12‐h light/dark cycle at a constant temperature of 26°C, with three rats per cage measuring 485 × 350 × 200 mm. The experimental procedures were performed in a Specific‐Pathogen‐Free (SPF) environment. After blood collection from the abdominal aorta, all rats were euthanized by cervical dislocation immediately following anesthesia with isoflurane to minimize animal suffering. The study was conducted with the approval of the Animal Care and Use Committee at Guangzhou University of Traditional Chinese Medicine (Permit Number 20240109005) and complied with the ARRIVE guidelines (Percie Du Sert et al. 2020).

The dose of ZYSD was designed based on daily doses of the six medicinal herbs prescribed by *Chinese Pharmacopeia*; designed dose of ZYSD in humans was 45 g (calculated as weight of mixed medicinal herbs that is, 5.00 g of ES, 2.50 g of ZO and GU, 1.67 g of PA, PC and SM per day)/60 kg (body weight) (Cp 2020). To evaluate its pharmacological effect, 0.5, 2.0, and 4.0 times human equivalent dose were set as low, middle, and high doses in rats.

**FIGURE 1:**
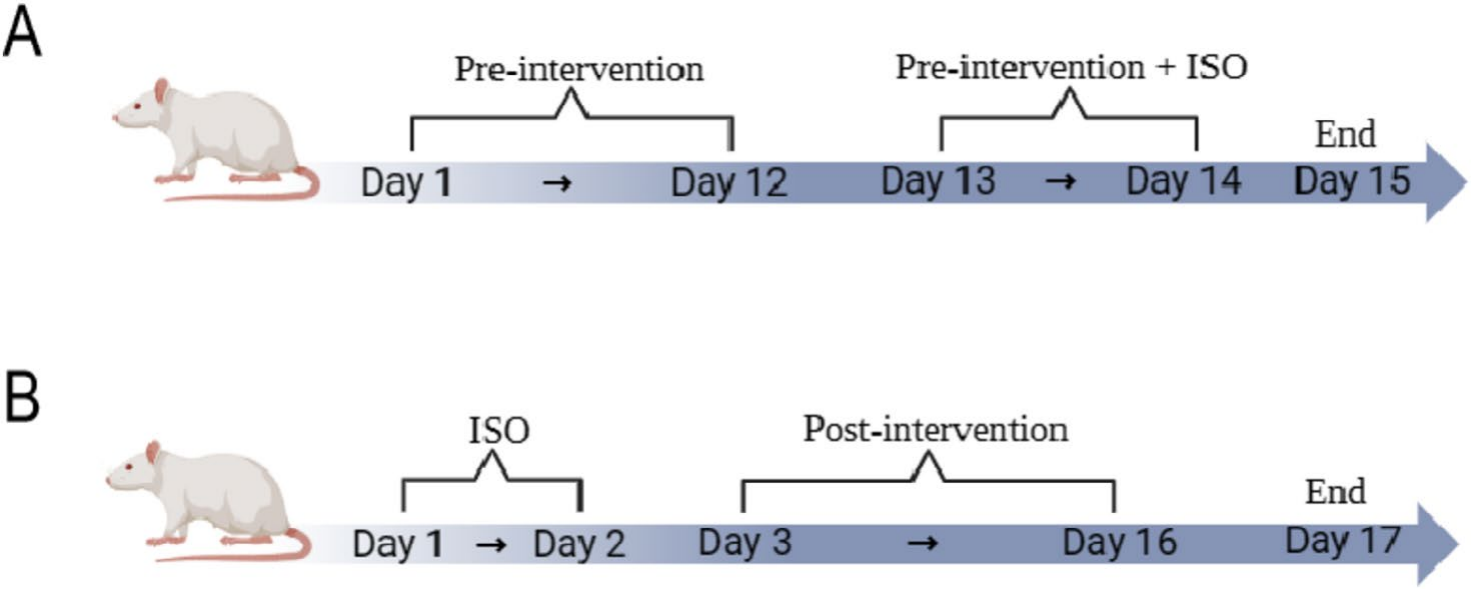
Experimental procedure schematic diagram. (A) Schematic diagram of pre‐intervention experiment with isoproterenol‐induced ischemic myocardial infarction. (B) Schematic diagram of post‐intervention experiment with isoproterenol‐induced ischemic myocardial infarction.

In the study evaluating the preventive effects of ZYSD on myocardial infarction induced by isoproterenol, 45 male rats, aged 8 weeks, were randomly assigned to the following groups: Pre‐normal control group (PreNC) consisting of 6 rats, Pre‐model control group (PreMC) with 9 rats, Pre‐low‐dose ZYSD group (PreLZ) comprising 7 rats, Pre‐medium‐dose ZYSD group (PreMZ) also with 7 rats, Pre‐high‐dose ZYSD group (PreHZ) including 8 rats, and Pre‐positive control metoprolol group (PrePC) with 8 rats. Throughout the intervention phase, the PreNC and PreMC groups received 10 mL/kg body weight of normal saline orally, while the PreLZ, PreMZ, and PreHZ groups were treated with ZYSD at dosages of 0.39, 1.57, and 3.14 g/kg (quantity of the mixed TCM decoction pieces/body weight), respectively. The PrePC group was administered metoprolol at a dosage of 10 mg/kg body weight. This treatment was administered daily for 14 consecutive days. On the 13th and 14th days, all groups except PreNC were subcutaneously injected with isoproterenol at a dosage of 85 mg/kg, which is consistent with a previous study (Abdullah‐Al‐Mamun et al. 2025). Following isoproterenol administration, the survival counts were as follows: PreNC, PreMC, PreHZ, and PrePC groups each had 6 surviving rats, while PreLZ and PreMZ groups each had 5 surviving rats (Figure 1A).

In the study examining the therapeutic effects of ZYSD on myocardial infarction induced by isoproterenol, 45 male rats, each 8 weeks of age, were randomly assigned to the following groups: Post‐Intervention Normal Control (PostNC, *n* = 4) and Modeling Group (*n* = 41). Isoproterenol was administered continuously at a dosage of 85 mg/kg on the first and second days of the experiment. The process resulted in the death of 14 rats, with successful modeling achieved in 27 rats. The post‐modeling group was further divided into Post‐Modeling Control (PostMC, *n* = 6), Post‐Low Dose (PostLZ, *n* = 5), Post‐Medium Dose (PostMZ, *n* = 5), Post‐High Dose (PostHZ, *n* = 6), and Post‐Propranolol Positive Control (PostPC, *n* = 5). From the third day of the experiment, intragastric administration was initiated, with the PostNC and PostMC groups receiving 10 mL/kg body weight of normal saline, and the PostLZ, PostMZ, and PostHZ groups, along with the PostPC group, receiving their respective treatments for 14 consecutive days (Figure 1B).

### 2.4 Cardiac Function Assessment

Anesthesia was induced in the rats by inhalation of 1% isoflurane. Subsequently, the chest hair of the rats was removed using Pure Hair Removal Cream (Veet, a product of Reckitt Benckise). Medical Ultrasonic Coupling Gel was then applied to the thoracic region of the rats to facilitate ultrasound transmission.

Cardiac function assessment was conducted using a high‐resolution small animal ultrasound system (model: Vevo, manufactured by Visual Sonics Co., LTD, based in Canada). The echocardiographic parameter measured included the end‐diastolic left ventricular internal diameter (LVEDD) and the end‐systolic left ventricular internal diameter (LVESD).

The fractional shortening (FS%) was derived from the formula FS% = (LVEDD − LVESD)/LVEDD × 100, which reflects the percentage change in the left ventricular diameter from diastole to systole. The left ventricular end‐diastolic volume (EDV) was calculated using the formula EDV = 7 × LVEDD^3^/(2.4 + LVEDD), and the left ventricular end‐systolic volume (ESV) was computed with the formula ESV = 7 × LVESD^3^/(2.4 + LVESD).

Finally, the ejection fraction (EF%), a key indicator of cardiac pumping efficiency, was determined by the equation EF (%) = (EDV − ESV)/EDV × 100. This parameter provides a percentage value representing the proportion of blood ejected from the left ventricle with each heartbeat. All measurements were performed in a standardized manner to ensure the accuracy and reliability of the collected data.

### 2.5 Electrocardiogram (ECG) Examination

Following the completion of the cardiac function tests, the leads of a small animal electrocardiogram (ECG‐3306B, Guangzhou 3ray Electronic Technology Co. Ltd.) were attached to the limbs and chest of the rats in accordance with the manufacturer's instructions for ECG data collection.

### 2.6 Collection of Cardiac Tissues

Following anesthesia induction with pentobarbital at a dosage of 50 mg/kg, the 1st to 7th left thoracic ribs were carefully excised with surgical scissors to gain access to the heart. The heart was then delicately dissected free from surrounding tissues, including the ascending aorta, pulmonary artery, pulmonary veins, and pericardium, using fine forceps and scissors. The heart was divided into two halves at the junction of the left and right ventricles. The left ventricle was further bisected, and the upper portion was fixed in 4% paraformaldehyde for subsequent pathological analysis. The lower portion was placed in a cryopreservation tube and stored in a −80°C freezer for future analysis.

### 2.7 Cardiac Pathological Examination

Fixed in a 4% paraformaldehyde solution (BIOSHARP, Labgic Technology Co. Ltd., China), the rat heart tissues were sent to Yuebin Pathology Laboratory for further analysis. There, the specimens underwent a standardized Hematoxylin and Eosin (H&E) staining protocol, as detailed in reference (Wu et al. 2022). Briefly, the fixed tissues were first dehydrated with 70%–100% gradient ethanol, then cleared with xylene to facilitate paraffin embedding. Paraffin blocks were prepared, and 5‐μm thick sections were cut using a microtome, followed by dewaxing and rehydration. Sections were stained with hematoxylin for nuclear labeling and eosin for cytoplasmic staining, then re‐dehydrated, cleared, and mounted with neutral balsam. Finally, the stained sections were observed under a light microscope for pathological assessment of myocardial tissue changes, ensuring consistent and reliable results.

### 2.8 Myocardial Assessment

Concentrations of TNF‐α, IL‐6, IL‐1β, Superoxide Dismutase (SOD), and Glutathione (GSH) in myocardial tissue were quantified using commercially available kits in accordance with the manufacturer's instructions, and the absorbance values were read using a microplate reader (Multiskan Mk3, Thermo Fisher (Shanghai) Instrument Co. Ltd). The ELISA kits employed were as follows: TNF‐α, IL‐6, IL‐1β (Servicebio, China, Lot Numbers: GER0004, GER0001, GER0002), SOD, GSH (Nanjing Jiancheng Bioengineering Institute, China, Lot Numbers: A001‐1‐1, A006‐2), thiobarbituric acid reactive substances (TBARS) and lipid hydroperoxides (LOOH) (Beijing Solarbio Science & Technology Co. Ltd., Cat: BC6 (Deng et al. 2022; Jiashuo et al. 2022) BC5245).

### 2.9 Serum Collection

After anesthesia induction with pentobarbital (50 mg/kg), an incision was made in the abdominal skin of the rats with scissors. Subsequently, the tissue overlying the abdominal aorta was gently separated using a gauze pad to fully expose the vessel. Blood was then collected from the abdominal aorta of the rats. Post‐collection, the blood samples were centrifuged at 3000× *g* for 15 min to separate the serum, and the separated serum was collected for subsequent analysis.

### 2.10 Serum Analysis

The serum concentrations of creatine kinase (CK) and its MB isoenzyme (CK‐MB) were determined utilizing commercially procured assay kits, strictly adhering to the protocols provided by the manufacturers. Quantification of these biomarkers was carried out using a microplate reader (model: Multiskan Mk3, manufactured by Thermo Fisher (Shanghai) Instrument Co. Ltd). The details of the ELISA kits used are as follows: CK (Redu Life Sciences Co. Ltd., China, Lot Number: S03024) and CK‐MB (Changchun Huili Biotechnology Co. Ltd., China, Lot Number: C060).

### 2.11 Fecal Collection and Analysis

After blood collection from the abdominal aorta, the cecum and its contents were collected from the rats and placed into 5‐mL cryopreservation tubes. The samples were immediately stored in −80°C freezer. Meanwhile, fecal samples were sent to Personalbio (Shanghai Personal Biotechnology Co. Ltd.) for further analysis. The detection methodology employed is detailed in a previously published article by our research group (Deng et al. 2022).

### 2.12 Network Pharmacology Processing Steps

The specific principles of network pharmacology can be referred to in the work by Jiashuo Wu (Jiashuo et al. 2022). In brief, the process entails retrieving the active ingredients present in TCM formulas and their corresponding targets (i.e., drug targets) from drug databases. Simultaneously, disease targets related to the studied condition are retrieved from disease databases. The intersection of drug targets and disease targets is then identified, which yields potential targets for the treatment of the disease by the active ingredients in the herbal formulation. The specific steps implemented in this study are summarized as follows:

#### 2.12.1 TCM Database Query

The Lab of Systems Pharmacology (https://old.tcmsp‐e.com/tcmspsearch.php) was used and input “ *Salvia Miltiorrhiza* ” into the “herb name” field. Within the “ingredients” section, molecular entities were filtered with an oral bioavailability (OB) ≥ 5 and drug‐likeness (DL) ≥ 0.18. In the “related targets” section, the molecular targets associated with the active components of *Salvia Miltiorrhiza* were extracted for subsequent analysis.

#### 2.12.2 Gene Name Acquisition

The UniProt website was used to identify the primary gene names corresponding to the molecular targets of *Salvia Miltiorrhiza* by entering the target names into the search bar and retrieving the results. A Python script was employed to integrate the molecular IDs with their corresponding gene names, and the integrated data was as a TXT document. Then, this process was repeated for other TCM components such as *Epimedium*, *Poria Cocos*, *Panax Ginseng*, *Zingiberis Rhizoma*, and *Glycyrrhiza*. Finally, the refined dataset of drug components and their corresponding target genes was imported into Cytoscape to construct a comprehensive docking diagram.

#### 2.12.3 Acquisition of Ischemic Myocardial Infarction‐Related Genes

The key word “myocardial infarction” was used to search the GeneCards database (https://www.genecards.org/), and a list of gene targets related to the condition was compiled from the search results. The retrieved results were filtered to retain only those genes with a relevance score ≥ 5, and filtered gene information was export in EXCEL format.

#### 2.12.4 Venn Diagram Construction

The Venny diagram tool (http://www.liuxiaoyuyuan.cn/) was utilized to construct a Venn diagram for visualizing the intersection of drug targets and disease targets. Drug targets and disease targets were separately input into the corresponding lists in the tool, and the Venn diagram was generate to intuitively display the overlapping the shared targets between the two sets.

#### 2.12.5 Protein–Protein Interaction Analysis

The STRING database (https://cn.string‐db.org/) was proceeded to for the analysis of protein‐protein interactions, and the intersecting targets were inputted into the “Multiple protein” search field and initiated the search. After setting the confidence score for display interactions to ≥ 0.900, the resulting TSV file was downloaded and then imported into Cytoscape. Node size and yellow‐to‐red color gradients were applied to visualize the degree of gene interactions, where larger and redder nodes indicated a higher level of connectivity among genes.

#### 2.12.6 Gene Enrichment Analysis

The Metascape website (https://metascape.org/) was used for Gene Enrichment Analysis. Briefly, the intersecting gene names were imported into the gene list field, selecting “custom analysis” for further processing. Upon completion of the analysis, we accessed the “enrichment” section and selected categories such as “GO Biological Processes,” “GO Cellular Components,” “GO Molecular Functions,” and “KEGG Pathway.” This allowed us to obtain a detailed analysis of the biological context and signaling pathways associated with the identified targets.

### 2.13 Molecular Docking

The structures of ZYSD active ingredients identified by HPLC were retrieved from the PubChem database (https://pubchem.ncbi.nlm.nih.gov/), optimize these structures using molecular mechanics methods, and then the RCSB PDB database (https://www.rcsb.org/) was used to retrieve the 3D crystal structure of each target. Then, AutoDock software 4.2.6 was used to analyze the molecular docking between active ingredients and targets. Finally, PyMol software 2.5.5 was used to visualize the binding modes between active ingredients and target proteins.

### 2.14 Molecular Dynamics (MD) Simulation

The LEaP program 3 in AMBER22 was employed to hydrogenate the system. Subsequently, using the Amber Tool, add a 10 Å rectangular TIP3P water box 4 to each model and at the same time neutralize the charge of each model by adding counterions (sodium or chloride ions). All MD simulations were conducted in AMBER22 by calling the GPU accelerated pmemd program. The AMBER FF14SB force field was used for protein and the GAFF2 force field was used for ligand. For the missing force field parameters in GAFF2, the antechamber program was used to supplement them. In addition, the RESP charge of the ligand was first calculated at the HF/6‐31G* level of Gaussian 16 to obtain the ESP charge and then subjected to restrictive fitting using the antechamber program. Each model underwent 3 rounds of optimization. First, models were restricted to the entire protein and ligand to only optimize solvent molecules, and all protein atoms were limited by a potential of 3000 kcal/mol·Å. Second, the protein skeleton and ligand molecules were limited, optimized the protein side chains, and minimized this stage when the protein skeleton was restricted (500 kcal/mol·Å). Finally, the protein energy was minimized for 4000 cycles without any restrictions. Afterwards, the temperature of each system was gradually increased from 0°K to 300°K through a 50 ps heating process in a canonical ensemble (NVT), followed by a 100 ps isothermal‐isobaric ensemble (NPT) to bring the system density back to approximately 1.0 g/cm^3^. Throughout the entire molecular dynamics process, the cutoff value for van der Waals and electrostatic interactions was set to 12 Å. The Langevin method and the Beeman algorithm were respectively used to control the temperature of the system and to analyze the Newtonian equations of motion of the system. The SHAKE algorithm was used to constrain bonds involving hydrogen atoms, with a time step of 2 fs for all. Based on the MD simulation results, the Molecular Mechanics/Generalized Born Surface Area (MM/GBSA) method was used to calculate the binding free energy between the compound and AKT1 protein by running the MMPBSA.py program of AmberTools22 suite.

### 2.15 Western Blotting Analysis

Total protein from cardiac tissues was extracted using RIPA Lysis Buffer (Beyotime, Beyotime Biotech Inc., China), with the addition of 1% protease and phosphatase inhibitor. Then, the loading buffer was added to each sample and heated to denature the protein. SDS‐PAGE gels were used to separate proteins, and the proteins were transferred onto PVDF membranes. After incubation with primary and secondary antibodies, bands were imaged using a chemiluminescence assay kit, and the ImageJ software was used to obtain the grayscale values of 3 bands for statistical analysis. Anti‐NFκB p65, anti‐p‐NFκB p65, anti‐PI3K, anti‐p‐PI3K, and anti‐β‐Actin were purchased from Abmart (Abmart Pharmaceutical Technology Co. Ltd., China); anti‐NRF2 and anti‐p‐AKT were purchased from Affinity (Affinity Biosciences, China); anti‐TLR4 and anti‐AKT were purchased from Proteintech (Proteintech Group Inc., China); Goat Anti‐Rabbit IgG H&L and Goat Anti‐Mouse IgG H&L (HRP) preadsorbed were purchased from Abcam (Abcam Limited, China).

### 2.16 Statistical Analysis

All results were analyzed using SPSS 23 software. Quantitative data were expressed as mean ± standard error of the mean (s.e.m.). For datasets showing normal distribution with satisfactory variance homogeneity, *p* values were determined using one‐way analysis of variance (ANOVA) with Fisher's least significant difference analysis. Otherwise, *p* values were determined using the Kruskal–Wallis test. Differences were considered statistically significant at *p* < 0.05. For categorical data, if the minimum frequency was not less than 5, the chi‐squared test was used. Otherwise, Fisher's exact test was employed.

## 3 Results

### 3.1 Analysis of ZYSD Extract

**FIGURE 2:**
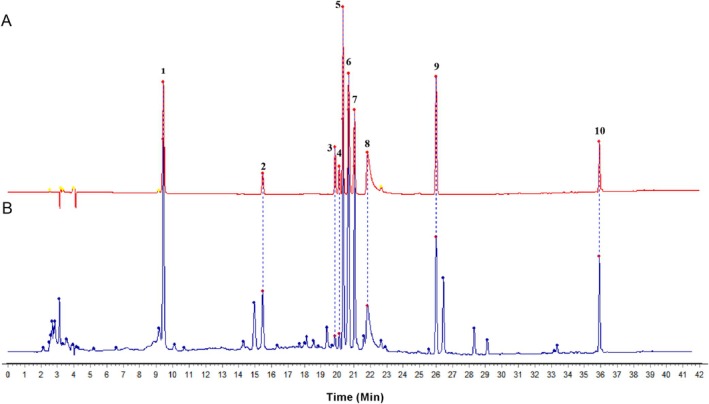
HPLC chromatograms for the reference compounds (A) and ZYSD (B). Identified plant constituent: 1‐liquiritin, 2‐ginsenosides Rb3, 3‐epimedin A, 4‐epimedin B, 5‐salvianolic acid C, 6‐epimedin C, 7‐icariin, 8‐quercetin, 9‐glycyrrhetinic acid, 10‐tanshinone IIA.

The HPLC fingerprint profiles of ZYSD were depicted in Figure 2. Comparing retention time (Supplementary Table 3) of the reference compound (Figure 2A) with the corresponding retention time of the compound peak recording in the HPLC chromatogram of ZYSD (Figure 2B), 10 marker constituents of medicinal herbs were identified. Specifically, peak 1 represents liquiritin, peak 2 as ginsenosides Rb3, peak 3 as epimedin A, peak 4 as epimedin B, peak 5 as salvianolic acid C, peak 6 as epimedin C, peak 7 as icariin, peak 8 as quercetin, peak 9 as glycyrrhetinic acid, peak 10 as tanshinone IIA (Figure 2). The HPLC fingerprint combined with the 10 identified marker components can be used for quality control of ZYSD.

### 3.2 ZYSD Prevention Improved Cardiac Function in ISO‐Induced Rats

**FIGURE 3:**
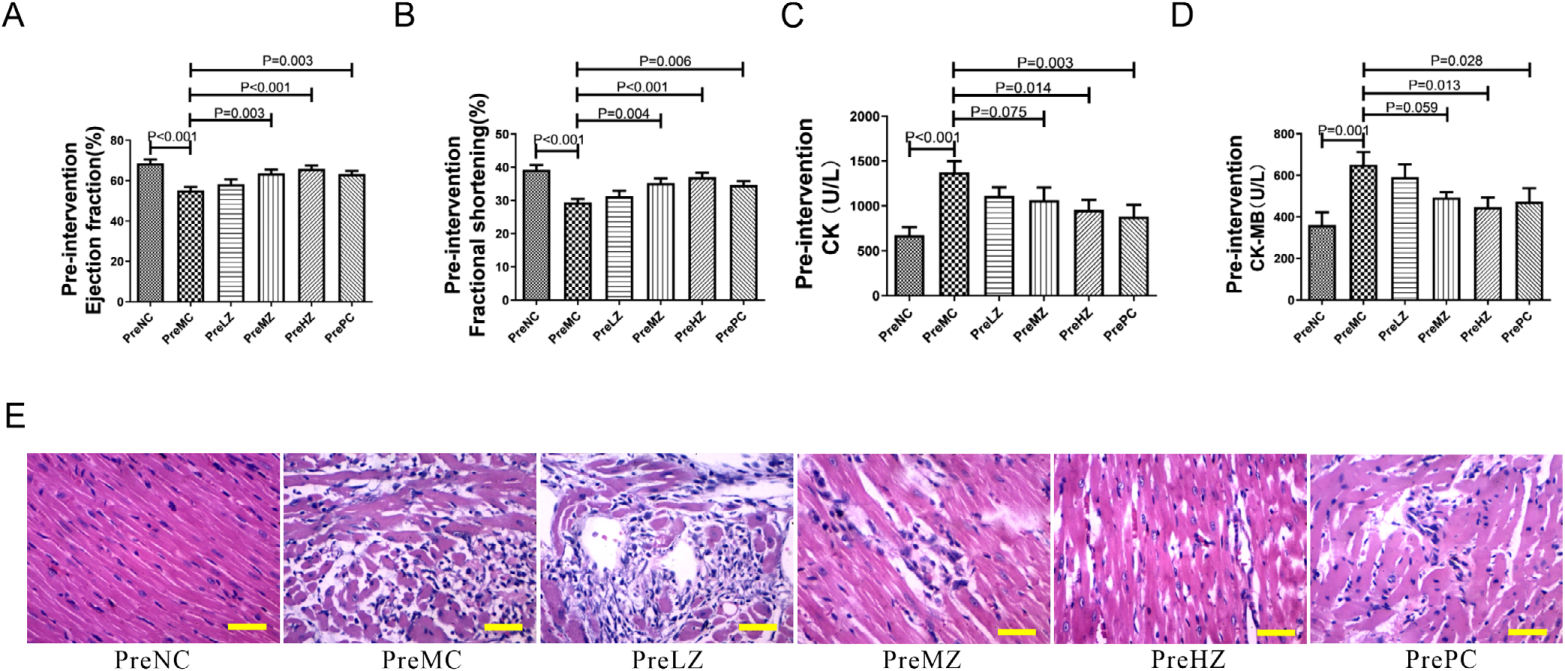
ZYSD prevention improved cardiac function in isoproterenol‐induced rats with ischemic myocardial infarction. (A) Ejection fraction of the rat hearts in the pre‐intervention experiment. (B) Fractional shortening of the rat hearts in the pre‐intervention experiment. (C) Levels of creatine kinase (CK) in the serum of rats in the pre‐intervention experiment. (D) Levels of creatine kinase‐MB (CK‐MB) in the serum of rats in the pre‐intervention experiment. (E) Representative images of myocardial sections stained with hematoxylin and eosin (H&E); the total magnification is 200×; scale bar = 100 μm. The *p* values were determined by one‐way analysis of variance (ANOVA) with Fisher's least significant difference analysis. Data presented as mean ± s.e.m. *n* = 6 for PreNC, PreMC, PreHZ, and PrePC; *n* = 5 for PreLZ and PreMZ.

As shown in Supplementary Table 4, pre‐intervention with ZYSD and metoprolol did not significantly reduce the mortality rate of isoproterenol‐induced myocardial infarction in rats. The ejection fraction and fractional shortening of rats in the pre‐intervention experiment are illustrated in the Figure 3A,B. Following intraperitoneal injection of isoproterenol, the ejection fraction and fractional shortening of the PreMC group were significantly lower than those of the PreNC group (Figure 3A,B). After pre‐intervention with medium‐dose ZYSD and high‐dose ZYSD, the ejection fraction and fractional shortening of rats in the PreMZ and PreHZ group were significantly higher than those in the PreMC group (Figure 3A,B). The ejection fraction and fractional shortening of rats in the PrePC group were also significantly higher than those in the PreMC group (Figure 3A,B). However, pre‐intervention with low dose of ZYSD did not significantly improve cardiac function. Also, ZYSD and metoprolol can both improve the electrocardiographic manifestations of acute myocardial infarction (Supplementary Figure 2). The pre‐intervention of ZYSD and metoprolol reduced the myocardial enzyme levels in acute myocardial infarction. Following intraperitoneal injection of isoproterenol, the CK and CK‐MB levels in the PreMC group were significantly higher than those in the PreNC group (Figure 3C,D), respectively. After intervention with ZYSD, the CK and CK‐MB levels in the rats of the PreMZ and PreHZ group were notably lower than those in the PreMC group (Figure 3C,D), respectively. Rats in the PrePC group also exhibited significantly lower CK and CK‐MB levels compared to the PreMC group (Figure 3C,D), respectively. The myocardial cells in the Pre‐model control group (PreMC) displayed extensive necrosis, characterized by significant dissolution, vacuolar degeneration, inflammatory cell infiltration, and atrophic myocardial cells (Figure 3E). ZYSD improved these pathological changes in a dose‐dependent manner; however, the improvement effect of metoprolol was not as good as ZYSD (Figure 3E). These results indicate that ZYSD prevention improves cardiac function in isoproterenol‐induced rats with ischemic myocardial infarction.

### 3.3 ZYSD Prevention Reduced the Levels of Inflammatory Factors and Oxidative Stress in ISO‐Induced Rats

**FIGURE 4:**
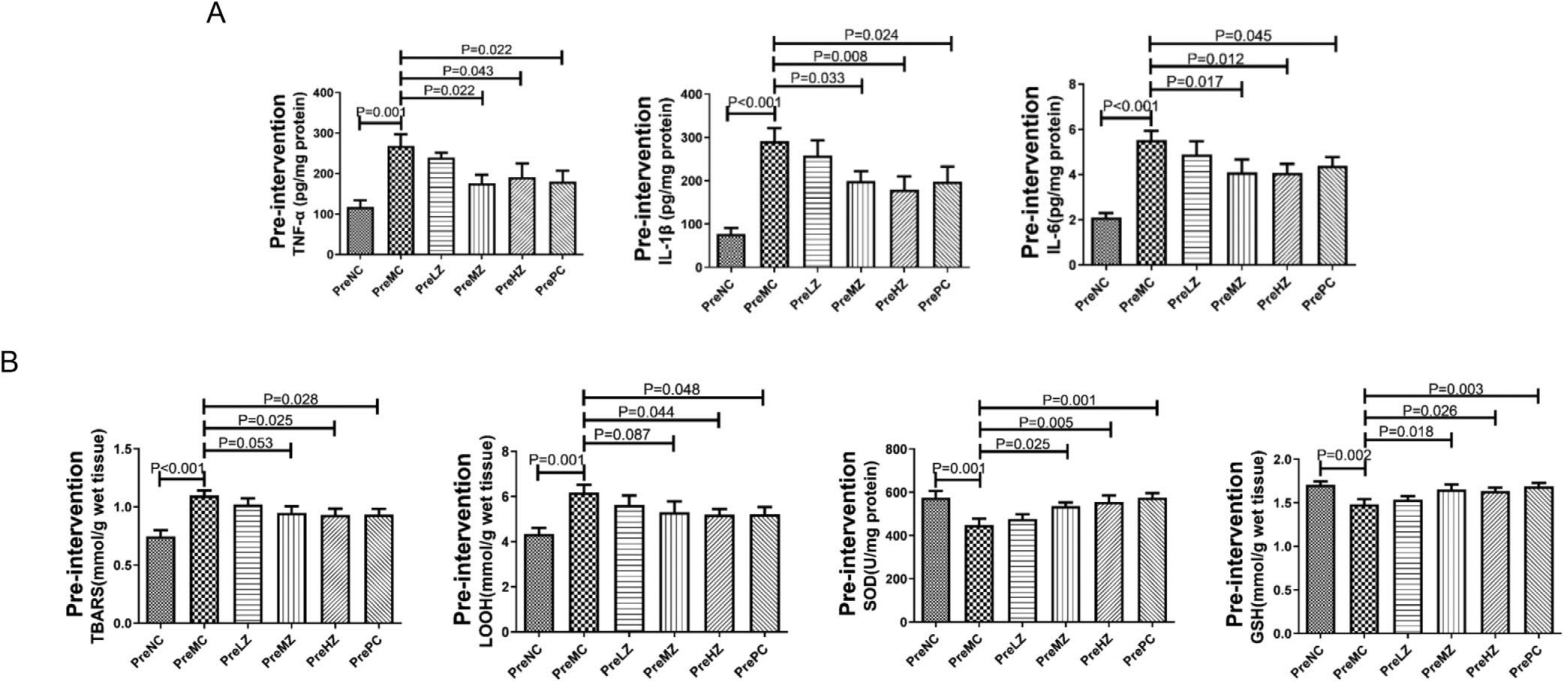
ZYSD prevention reduced inflammation and oxidative stress in isoproterenol‐induced rats with ischemic myocardial infarction. (A) Levels of TNF‐α, IL‐1β, and IL‐6 in the myocardium of rats. (B) Levels of TBARS, LOOH, SOD, and GSH in the rat myocardium. The *p* values were determined by one‐way analysis of variance (ANOVA) with Fisher's least significant difference analysis. Data presented as mean ± s.e.m. *n* = 6 for PreNC, PreMC, PreHZ, and PrePC; *n* = 5 for PreLZ and PreMZ.

Both ZYSD and metoprolol reduced the levels of inflammatory factors in rats after acute myocardial infarction. Following intraperitoneal injection of isoproterenol, the TNF‐α, IL‐1β, and IL‐6 levels in the PreMC group were significantly higher than those in the PreNC group (Figure 4A), respectively. After pre‐intervention with ZYSD, the rats in the PreMZ and PreHZ group showed significantly lower TNF‐α, IL‐1β, and IL‐6 levels compared to the PreMC group (Figure 4A). Rats in the PrePC group also exhibited significantly lower TNF‐α, IL‐1β, and IL‐6 levels than the PreMC group (Figure 4A). Both ZYSD and metoprolol improved the oxidative stress levels in acute myocardial infarction. Following intraperitoneal injection of isoproterenol, the TBARS and LOOH levels in the PreMC group were significantly higher than those in the PreNC group (Figure 4B), respectively. After pre‐intervention with ZYSD, the TBARS and LOOH levels in the rats in the PreMZ and PreHZ group were significantly lower than those in the PreMC group (Figure 4B). Rats in the PrePC group also exhibited significantly lower TBARS and LOOH levels compared to the PreMC group (Figure 4B). The SOD and GSH levels in the PreMC group were significantly lower than those in the PreNC group (Figure 4B). After ZYSD pre‐intervention, the rats in the PreMZ and PreHZ group showed significantly higher SOD and GSH levels compared to the PreMC group (Figure 4B). Rats in the PrePC group also exhibited significantly higher SOD and GSH levels than the PreMC group (Figure 4B). These results indicate that ZYSD prevention reduces inflammation and oxidative stress in isoproterenol‐induced rats with ischemic myocardial infarction.

### 3.4 ZYSD Treatment Improved Cardiac Function in ISO‐Induced Rats

**FIGURE 5:**
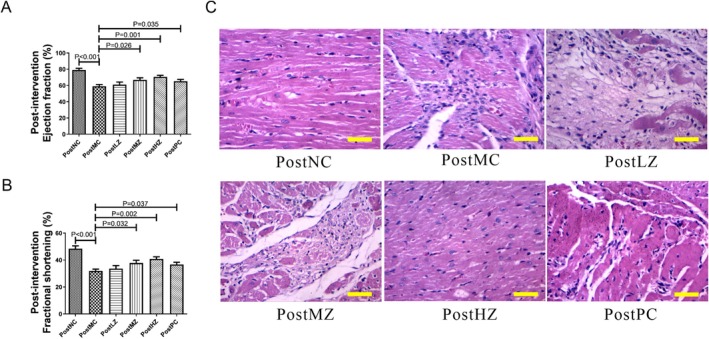
ZYSD treatment improved cardiac function in isoproterenol‐induced rats with ischemic myocardial infarction. (A) Ejection fraction of the rat hearts in the pre‐intervention experiment. (B) Fractional shortening of the rat hearts in the pre‐intervention experiment. (C) Representative images of myocardial sections stained with hematoxylin and eosin (H&E); the total magnification is 200×; scale bar = 100 μm. The *p* values were determined by one‐way analysis of variance (ANOVA) with Fisher's least significant difference analysis. Data presented as mean ± s.e.m. *n* = 4 for PostNC, *n* = 5 for PostMC, PostLZ, PostMZ, and PostPC; *n* = 6 for PostHZ.

The cardiac function of rats in the post‐intervention experiment is presented in Figure 5A–C. Following successful modeling, the intervention was applied to each group. The ejection fraction and fractional shortening in the PostMC group were significantly lower than those in the PostNC group (Figure 5A,B). After medium‐dose ZYSD and high‐dose ZYSD intervention, both ejection fraction and fractional shortening in the PostMZ, PostHZ group were significantly higher than those in the PostMC group (Figure 5A,B). Ejection fraction and fractional shortening of rats in the PostPC group were also significantly higher compared to the PostMC group (Figure 5A,B). However, low doses of ZYSD post‐intervention did not significantly improve cardiac function. Also, ZYSD and metoprolol can both improve the electrocardiographic manifestations of acute myocardial infarction (Supplementary Figure 3). Similar to the preventive effect, ZYSD improved these pathological changes in a dose‐dependent manner; the improvement effect of metoprolol was not as good as ZYSD (Figure 5C). These results indicate that ZYSD treatment improves cardiac function in isoproterenol‐induced rats with ischemic myocardial infarction.

### 3.5 ZYSD Treatment Reduced the Levels of Cardiac Enzymes in ISO‐Induced Rats

**FIGURE 6:**
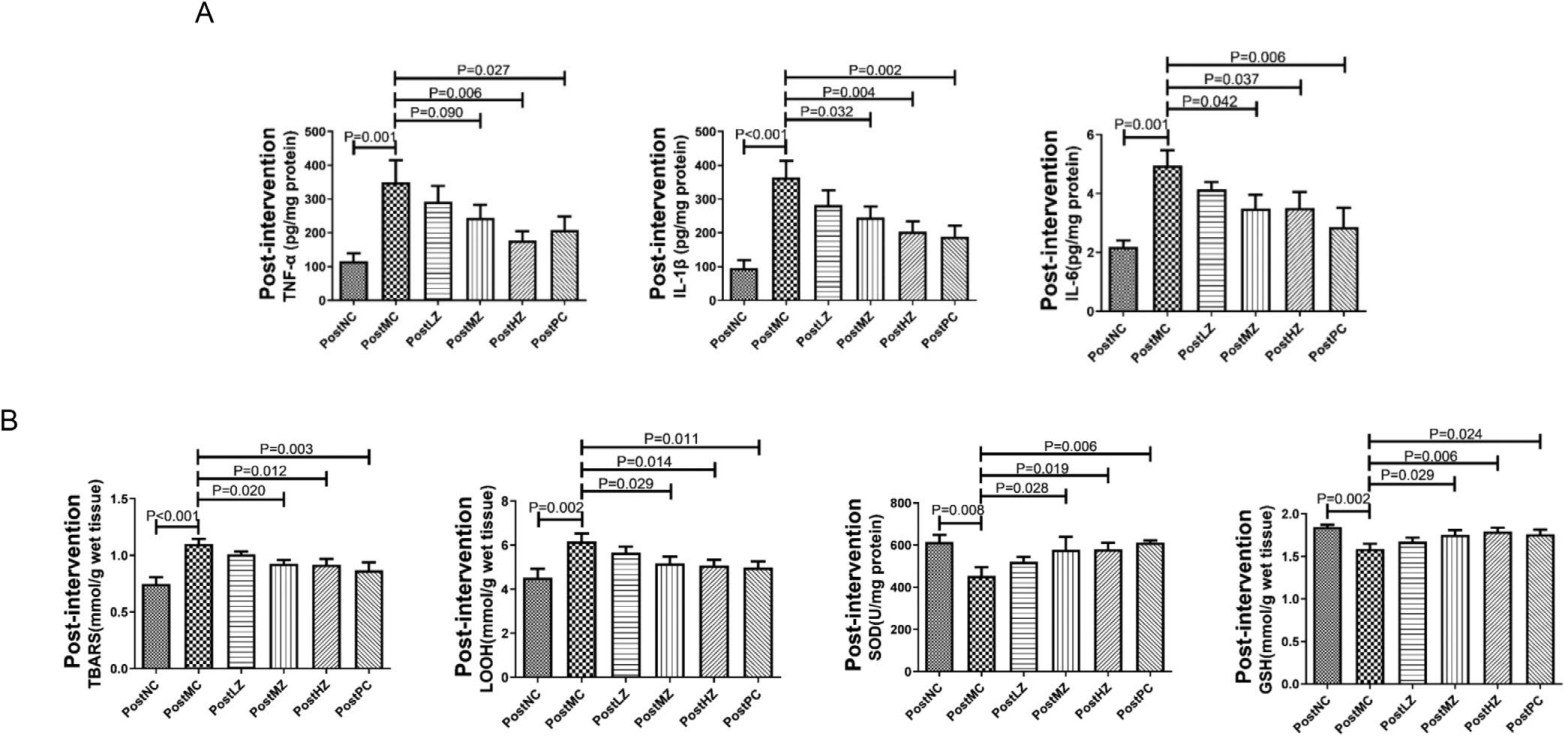
ZYSD treatment reduced inflammation and oxidative stress in isoproterenol‐induced rats with ischemic myocardial infarction. (A) Levels of TNF‐α, IL‐1β, and IL‐6 in the myocardium of rats. (B) Levels of TBARS, LOOH, SOD, and GSH in the rat myocardium. The *p* values were determined by one‐way analysis of variance (ANOVA) with Fisher's least significant difference analysis. Data presented as mean ± s.e.m. *n* = 4 for PostNC, *n* = 5 for PostMC, PostLZ, PostMZ, and PostPC; *n* = 6 for PostHZ.

Following successful modeling, corresponding interventions were administered to each group. The TNF‐α, IL‐1β, and IL‐6 levels in the PostMC group were significantly higher than those in the PostNC group (Figure 6A), respectively. After intervention with ZYSD, the TNF‐α, IL‐1β, and IL‐6 levels in the PostMZ and PostHZ group rats were notably lower than those in the PostMC group (Figure 6A), respectively. Rats in the PostPC group also exhibited significantly lower TNF‐α, IL‐1β, and IL‐6 levels compared to the PostMC group (Figure 6A), respectively. The TBARS and LOOH levels in the PostMC group were significantly higher than those in the PostNC group (Figure 6B), respectively. After intervention with ZYSD, the TBARS and LOOH levels in the rats of the PostMZ and PostHZ group were notably lower than those in the PostMC group (Figure 6B), respectively. Rats in the PostPC group also exhibited significantly lower TBARS and LOOH levels compared to the PostMC group (Figure 6B), respectively. Additionally, the SOD and GSH levels in the PostMC group were significantly lower than those in the PostNC group (Figure 6B), respectively. Following ZYSD intervention, the SOD and GSH levels in the rats of the PostMZ and PostHZ group were significantly higher than those in the PostMC group (Figure 6B), respectively. Rats in the PostPC group also exhibited significantly higher SOD and GSH levels compared to the PostMC group (Figure 6B), respectively. These results indicate that ZYSD treatment reduces inflammation and oxidative stress in isoproterenol‐induced rats with ischemic myocardial infarction.

### 3.6 ZYSD Affected Fecal Related Abundance of *Lactobacillus*, *Bifidobacterium* and *Clostridium* in ISO‐Induced Rats

**FIGURE 7:**
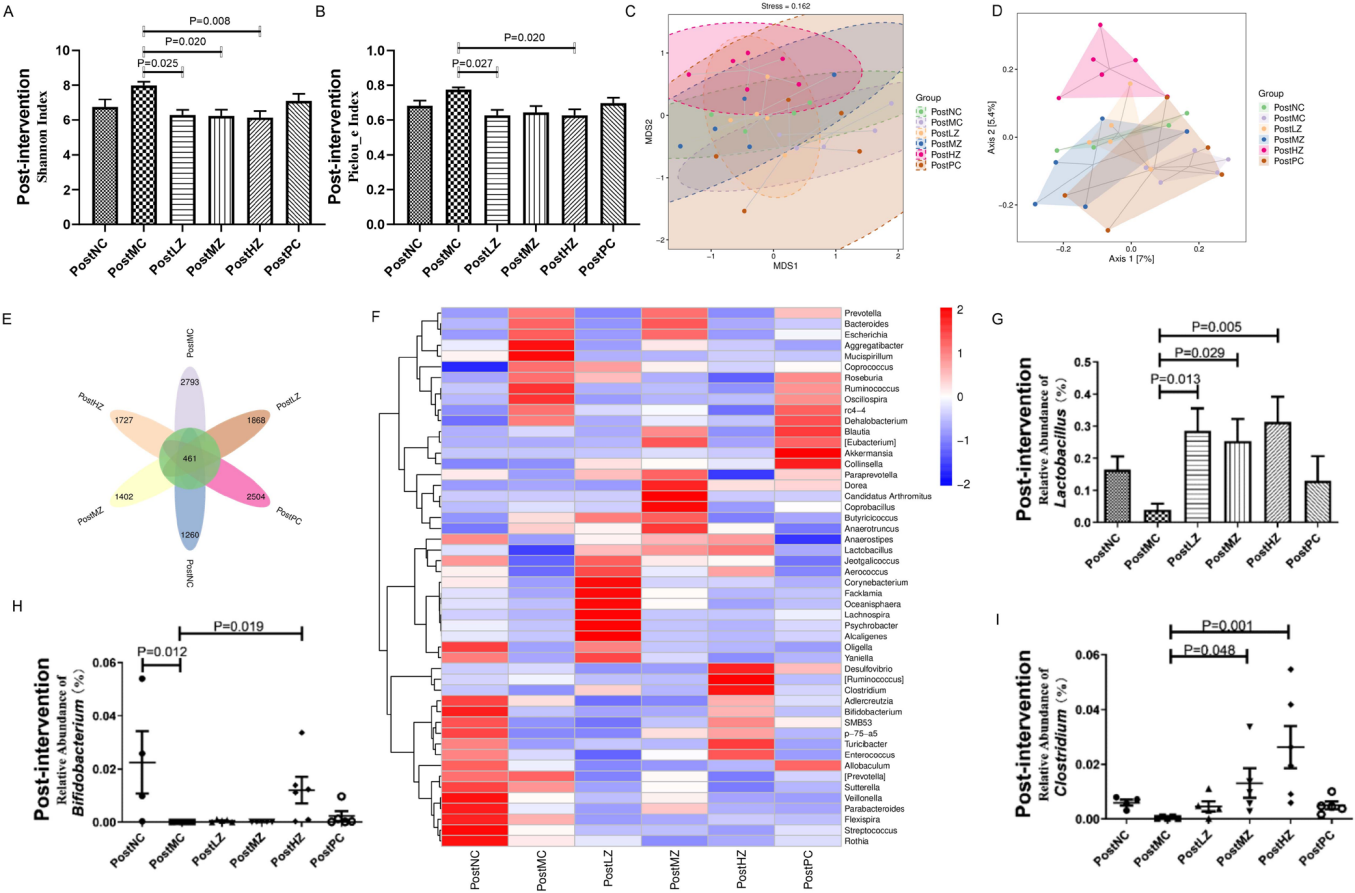
ZYSD treatment up‐regulated beneficial bacterial genera that contribute to the improvement of ischemic myocardial infarction. (A) Shannon index. (B) Pielou_e index. (C) Principal co‐ordinates analysis (PCoA). (D) Non‐metric multidimensional scaling (NMDS) analysis. (E) Flower plot. (F) Species composition heatmap in genus level. (G) The relative abundance of *Lactobacillus* in the rat feces. (H) The relative abundance of *Bifidobacterium* in the rat feces. (I) The relative abundance of *Clostridium* in the rat feces. For (G), the *p* values were determined by one‐way analysis of variance (ANOVA) with Fisher's least significant difference analysis. Data presented as mean ± s.e.m. For (H) and (I), the *p* values were determined using the Kruskal–Wallis test. Each point represents an individual mouse. *n* = 4 for PostNC, *n* = 5 for PostMC, PostLZ, PostMZ, and PostPC; *n* = 6 for PostHZ.

After successful modeling, each group underwent corresponding interventions. Shannon index and Pielou_e index were used to evaluate the alpha diversity of flora. Samples in the PostMC group showed a higher Shannon index and Pielou_e index compared with those in the PostNC group (Figure 7A,B). ZYSD effectively recovered the Shannon index and Pielou_e index, while metoprolol had no improvement on them (Figure 7A,B). Principal Co‐ordinates Analysis (PCoA) and Non‐metric Multidimensional Scaling (NMDS) analysis were used to evaluate the beta diversity of flora, *p* = 0.001 (Figure 7C,D). A total of 461 ASV/OTU had been found among 6 groups (Figure 7E). The abundance data of the top 50 genera with average abundance were used to draw a heatmap, and significant intergroup differences were observed (Figure 7F). ZYSD affected the relative abundance of *Lactobacillus*, *Bifidobacterium*, and *Clostridium* in feces. The fecal relative abundance of *Lactobacillus* in the PostLZ, PostMZ, and PostHZ groups was significantly higher than in the PostMC group (Figure 7G). The fecal relative abundance of *Bifidobacterium* in the PostMC group was significantly lower than in the PostNC group (Figure 7H). The fecal relative abundance of *Bifidobacterium* in the PostHZ group was significantly higher than in the PostMC group (Figure 7H). The fecal relative abundance of *Clostridium* in the PostHZ and PostMZ groups was significantly higher than in the PostMC group (Figure 7I). These results indicate that ZYSD treatment up‐regulated beneficial bacterial genera that contribute to the improvement of ischemic myocardial infarction.

### 3.7 Network Pharmacology Analysis of Potential Compounds in ZYSD for Ischemic Myocardial Infarction

**FIGURE 8:**
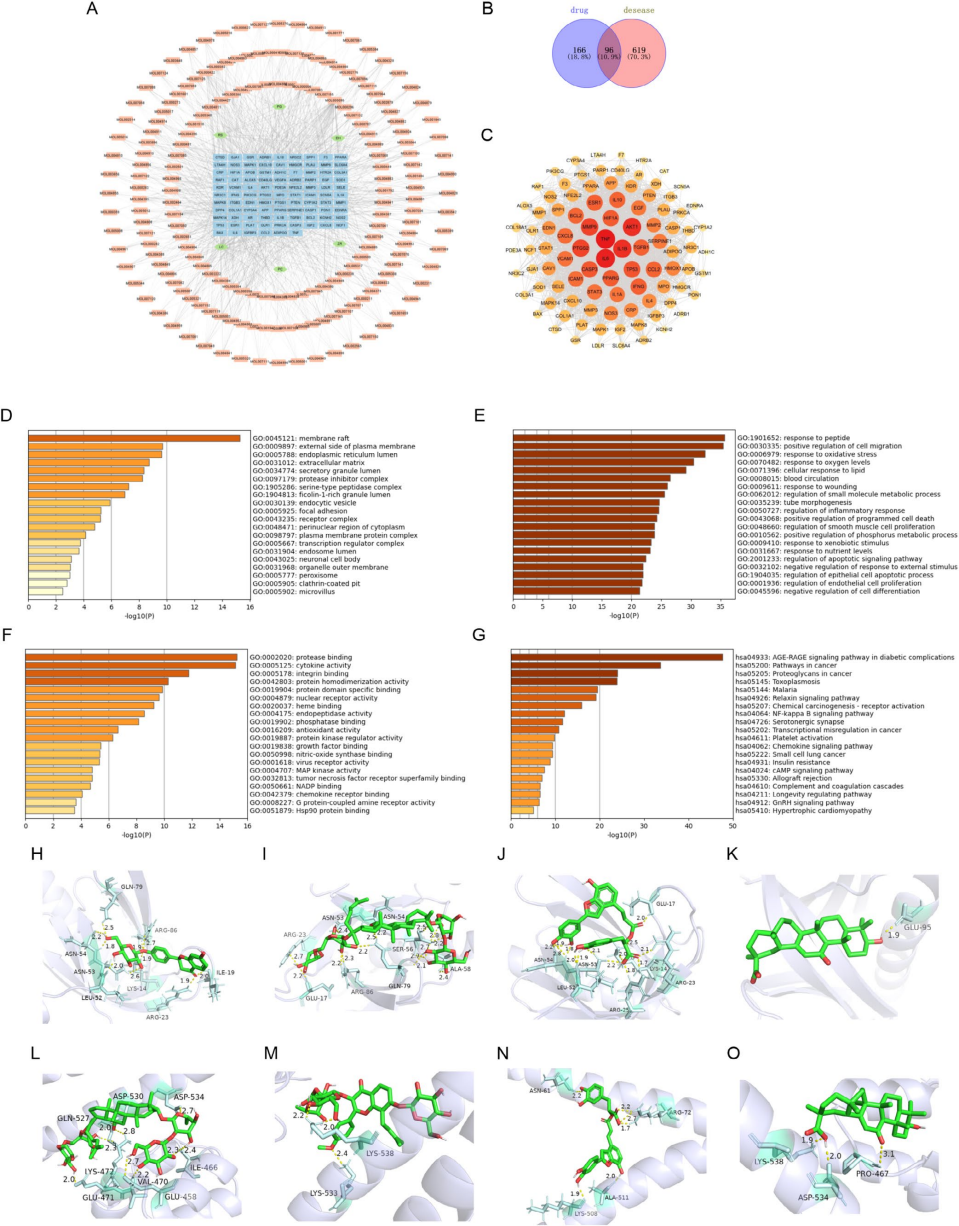
Network pharmacology and molecular docking analysis. (A) ZYSD active ingredient‐target network. (B) Venn diagram of active ingredients and disease targets. (C) The potential target protein interaction map of ZYSD, and the closer its color is to red, the more related targets interact with that target. (D) Cellular compound. (E) Biological process. (F) Molecular function. (G) KEGG enrichment analysis. (H) Molecular docking between liquiritin and AKT1. (I) Molecular docking between ginsenosides Rb3 and AKT1. (J) Molecular docking between salvianolic acid C and AKT1. (K) Molecular docking between glycyrrhetinic acid and AKT1. (L) Molecular docking between ginsenosides Rb3 and NRF2. (M) Molecular docking between epimedin A and NRF2. (N) Molecular docking between salvianolic acid C and NRF2. (O) Molecular docking between glycyrrhetinic acid and NRF2.

From the database, a total of 421 compounds present in ZYSD were collected. There are 262 target proteins affected by these compounds. Ischemic myocardial infarction has 715 disease‐related target proteins. As illustrated in the network pharmacology diagram (Figure 8), there are 96 intersecting target proteins between the drug targets and disease‐related targets. This suggests that ZYSD might intervene in ischemic myocardial infarction through these potential 96 target proteins (Figure 8A). After analyzing the intersection of the aforementioned 96 potential targets using the STRING 11.0 database, a protein–protein interaction analysis graph (Figure 8C) was generated, consisting of 96 nodes and 230 edges. The main components and targets of ZYSD in alleviating ischemic myocardial infarction are shown as Supplementary Table 5.

After uploading the aforementioned 96 targets to the database, a GO enrichment analysis was conducted. “Membrane raft,” “External side of plasma membra,” “endoplasmic reticulum lumen,” and so on were obtained in Cellular Compound prediction (Figure 8D). “Response to peptide,” “response to oxidative stress,” “response to oxygen levels,” and so on were obtained in Biological Processes prediction (Figure 8E). “Protease binding”, “cytokine activity”, “integrin binding” and so on were obtained in Molecular Function prediction (Figure 8F). After uploading the aforementioned 96 targets to the database, a KEGG enrichment analysis was conducted. In the prediction, pathways such as “cAMP signaling pathway,” “Complement and coagulation cascades,” and “Hypertrophic cardiomyopathy” were identified (Figure 8G). These pathways are closely associated with the pathological mechanisms of ischemic myocardial infarction.

The ZYSD compounds detected by HPLC were selected to dock with the potential targets screened by network pharmacology, and the docking results of the four molecules with the best binding were displayed, docking with AKT1 and NRF2 respectively. As shown in Figure 8H, liquiritin docking to AKT1 had well combined with protein residues ASN‐54, ASN‐53, LEU‐52, LYS‐14, ARG‐23, ARG‐86, GLN‐79, ILE‐19, and the binding energy was −7.96 kcal/mol. As shown in Figure 8I, ginsenosides Rb3 docking to AKT1 had well combined with protein residues ARG‐23, ARG‐86, ASN‐53, ASN‐54, SER‐56, GLU‐17, GLN‐79, ALA‐58; the binding energy was −7.90 kcal/mol. As shown in Figure 8J, salvianolic acid C docking to AKT1 had well combined with protein residues GLU‐17, ASN‐54, ASN‐53, LEU‐52, ARG‐25, ARG‐23, LYS‐14; and the binding energy was −10.24 kcal/mol. As shown in Figure 8K, glycyrrhetinic acid docking to AKT1 had well combined with protein residues GLU‐95; and the binding energy was −7.5 kcal/mol. As shown in Figure 8L, ginsenosides Rb3 docking to NRF2 had well combined with protein residues GLN‐527, ASP‐530, ASP‐534, IYS‐472, GLU‐471, VAL‐470, GLU‐458, ILE‐466; and the binding energy was −7.70 kcal/mol. As shown in Figure 8M, epimedin A docking to NRF2 had well combined with protein residues LYS‐533, LYS‐538; and the binding energy was −6.50 kcal/mol. As shown in Figure 8N, salvianolic acid C docking to NRF2 had well combined with protein residues ASN‐61, ARG‐72, ALA‐511, LYS‐508; and the binding energy was −7.67 kcal/mol. As shown in Figure 8O, glycyrrhetinic acid docking to NRF2 had well combined with protein residues LYS‐538, ASP‐534, PRO‐467; and the binding energy was −6.27 kcal/mol. These results indicate that compounds of ZYSD bind well to AKT1 and NRF2, which is closely related to their effect in improving inflammation and oxidative stress.

### 3.8 MD Simulations Showed Stable Combination Between Compounds of ZYSD and AKT1

**FIGURE 9:**
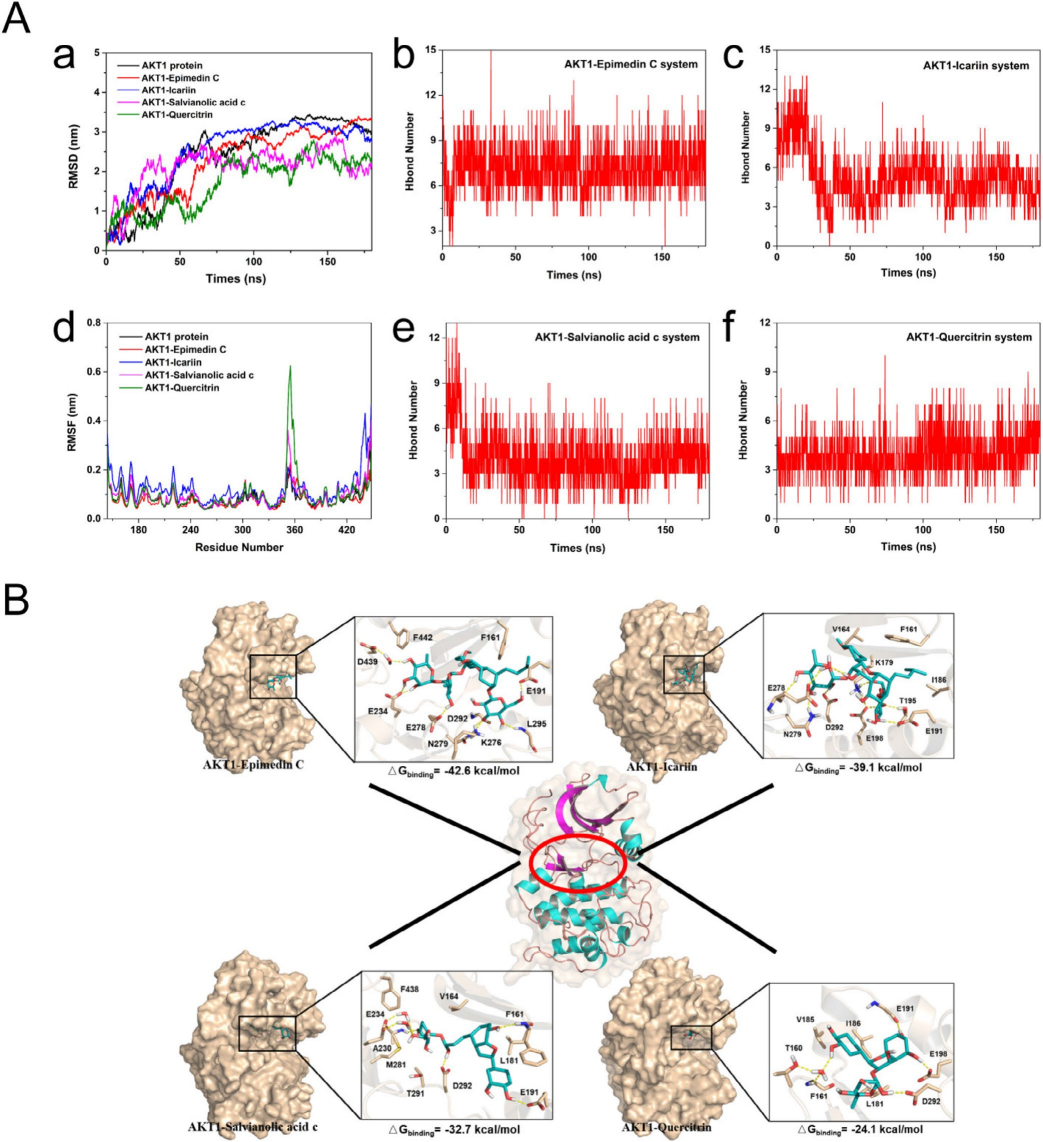
Molecular dynamic simulation analysis. (A) Molecular dynamics simulation analyses for AKT1 protein, AKT1‐Epimedin C, AKT1‐Icariin, AKT1‐Salvianolic acid C, and AKT1‐Quercitrin complex model. (a) Root‐mean‐square deviations (RMSD) of the heavy atoms in AKT1 protein (black), AKT1‐Epimedin C (red), AKT1‐Icariin (blue), AKT1‐Salvianolic acid C (magenta), and AKT1‐Quercitrin (green) model. (b) Root‐mean‐square fluctuations (RMSF) of the residues in AKT1 protein (black), AKT1‐Epimedin C (red), AKT1‐Icariin (blue), AKT1‐Salvianolic acid C (magenta), and AKT1‐Quercitrin (green) model. (c–f) The hydrogen bond number of Epimedin C (c), Icariin (d), Salvianolic acid C (e), and Quercitrin (f) with AKT1 protein in each complex model. (B) Representative structures of Epimedin C (top left), Icariin (top right), Salvianolic acid C (bottom left), and Quercitrin (bottom right) combined with AKT1 protein during MD simulations.

To investigate the binding mode and stability of ZYSD compounds with AKT1 protein, inclduing epimedin C, icariin, salvianolic acid C, and quercillin, the MD simulations were conducted, respectively (Figure 9A). The RMSD trend of protein and complex systems is similar, reaching dynamic equilibrium at approximately 80 ns. The average RMSD values of AKT1, Epimidin C‐AKT1, Icariin AKT1, Salvianolic acid c‐AKT1, and Quercitrin AKT1 systems are 2.43 ± 0.98 nm, respectively, 2.37 ± 0.89 nm, 2.53 ± 0.80 nm, 2.12 ± 0.49 nm, 1.71 ± 0.63 nm. There is not much difference in the fluctuation of RMSF values among different systems. For the structural domains Y350‐L362, Salvianolic acid c‐AKT1, and Quercitrin AKT1 that are far from the active pocket and adjacent to water, the fluctuation of residues in the two systems is intensified, which may be due to the effect of solvents. According to the hydrogen bond network diagram of each protein and compound, it was found that the compound can form at least 2–3 hydrogen bonds with the amino acids in the protein pocket, which play an important role in stabilizing the binding of the compound to AKT1 protein. Subsequently, composite structures at 100 ns were selected as the research objects for each system (Figure 9B). For the epimidin C‐AKT1 complex system, epimedin C binds to the ATP binding pocket of AKT1 protein, and its 3 glycosides form 8 polar interactions (or water mediated polar interactions) with the side chains of residues D439, E234, E278, N279, D292, K276, E191 and the main chain of residue L295, respectively. These hydrogen bond interactions greatly promote the binding of epimedin C, which is consistent with the hydrogen bond analysis results; In addition, epimidin C exhibits CH/π or hydrophobic interactions with the fatty chains of residues F144, F442, and E191, further enhancing the stability of compound binding. In the icarin‐AKT1 complex structure, similar to Epimidin C, the glycosyl portion has hydrogen bonding interactions with the side chains of protein residues E278, N279, D292, and E191, respectively. In addition, E198, T195, and K179 residues also have polar contacts with the compound. Icariin has CH/π interactions or hydrophobic interactions with hydrophobic residues V164, F161, and I186 around the pocket, which facilitates more stable binding of the compound to AKT1 protein. For the salvianolic acid c‐AKT1 complex system, the hydroxyl groups of the compound form hydrogen bonding (or water mediated) interactions with protein residues E234, A230, M281, D292, E191, and F161, respectively, and have hydrophobic interactions with pocket hydrophobic residues F438, V164, L181, and F161 benzene rings. In the Quericin‐AKT1 complex structure, polar interactions were formed between the compound and residues T160, F161, D292, E198, and E191, while residues V185, I186, and L181 stabilized the binding of the compound through hydrophobic interactions. Furthermore, The combined free energies of these complexes were −42.6 ± 0.5, −39.1 ± 0.5, −32.8 ± 0.3, and −24.2 ± 0.3 kcal/mol (Supplementary Table 6). These results indicate that compounds of ZYSD bind stably to AKT1.

### 3.9 Both ZYSD Prevention and Treatment Regulated PI3K/AKT1/NRF2 Signaling

**FIGURE 10:**
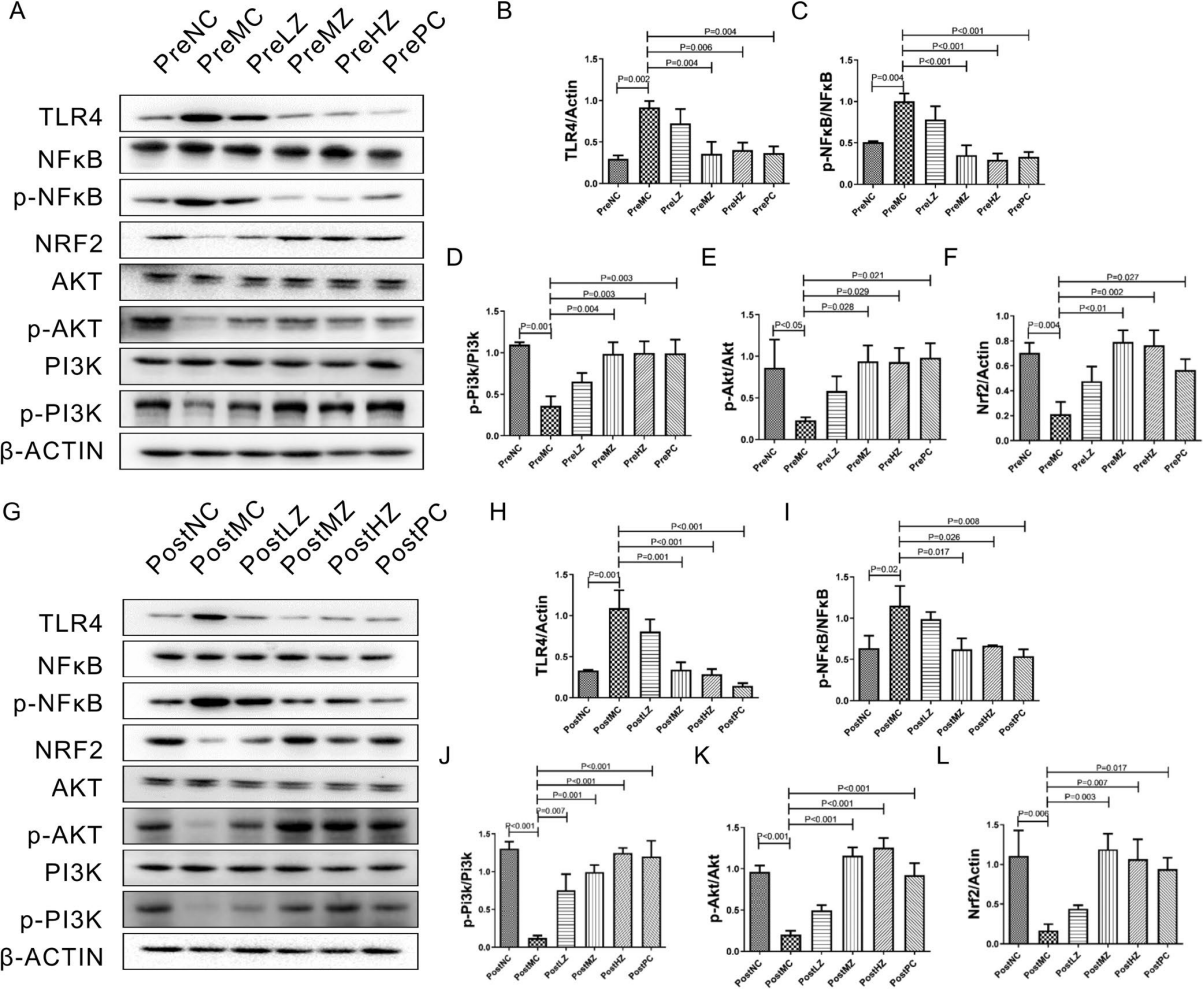
ZYSD prevention and treatment regulated TLR4/NFκB and PI3K/AKT1/NRF2 signaling pathway. (A–F) Western blotting analysis of TLR4, NFκB, p‐NFκB, NRF2, AKT, p‐AKT, PI3K, and p‐PI3K in ZYSD prevention experiment. (G–L) Western blotting analysis of TLR4, NFκB, p‐NFκB, NRF2, AKT, p‐AKT, PI3K, and p‐PI3K in ZYSD treatment experiment. β‐Actin was used as an internal control. The *p* values were determined by one‐way analysis of variance (ANOVA) with Fisher's least significant difference analysis. Data presented as mean ± s.e.m. *n* = 3.

After intraperitoneal injection of isoproterenol, TLR4 and NFκB levels in the PreMC group were significantly higher than those in the PreNC group (Figure 10A–F). After pre‐intervention with ZYSD, TLR4 and NFκB in the PreMZ and PreHZ groups were significantly lower than those in the PreMC group (Figure 10A–F). TLR4 and NFκB of rats in the PrePC group were significantly lower than those in the PreMC group (Figure 10A–F). After intraperitoneal injection of isoproterenol, the levels of PI3K, Akt, and Nrf2 in the PreMC group were significantly lower than those in the PreNC group (Figure 10A–F). Following pre‐intervention with ZYSD, the levels of PI3K, Akt, and Nrf2 in the PreMZ and PreHZ groups were significantly higher than those in the PreMC group (Figure 10A–F). Moreover, in the PrePC group, the levels of PI3K, Akt, and Nrf2 in rats were also significantly higher than those in the PreMC group (Figure 10A–F).

After successful modeling, corresponding interventions were performed for each group, and TLR4 and NFκB levels in the PostMC group were significantly higher than those in the PostNC group (Figure 10G–L). After pre‐intervention with ZYSD, TLR4 and NFκB in the PostMZ and PostHZ groups were significantly lower than those in the PostMC group (Figure 10G–L). TLR4 and NFκB in the PostPC group were also significantly lower than those in the PostMC group (Figure 10G–L). The levels of PI3K, Akt, and Nrf2 in the PostMC group were significantly lower than those in the PostNC group (Figure 10G–L). Following ZYSD intervention, the rats in the PostMZ and PostHZ groups exhibited significantly higher levels of PI3K, Akt, and Nrf2 compared to the PostMC group (Figure 10G–L). Moreover, rats in the PrePC group also showed significantly higher levels of PI3K, Akt, and Nrf2 compared to the PostMC group (Figure 10G–L).

These results indicate that ZYSD prevention and treatment regulate the PI3K/AKT1/NRF2 signaling pathway.

## 4 Discussion

This study aimed to explore the preventive and therapeutic effects of ZYSD on isoproterenol‐induced myocardial infarction. In the investigation of ZYSD's preventive effects on isoproterenol‐induced myocardial infarction, medium and high‐dose ZYSD were found to reduce infarct size and improve cardiac function in the rat model of isoproterenol‐induced myocardial infarction, similar to those of clinically used metoprolol tartrate. Similar outcomes were observed in the investigation of ZYSD's therapeutic effects on isoproterenol‐induced myocardial infarction, which were consistent with the results obtained in the preventive experiments.

To ensure experimental reproducibility, an HPLC fingerprint combined with the identification of 10 marker components was established for the quality control of ZYSD. This approach also indicated the potential effect of ZYSD's marker components, including epimedin C, icariin, epimedin A, epimedin B, liquiritin, glycyrrhetinic acid, quercetin, ginsenosides Rb3, salvianolic acid C and tanshinone IIA. These components are also bioactive component of *Epimedium*, *Panax ginseng*, *Salvia Miltiorrhiza* and *Glycyrrhiza* (Liu et al. 2023). However, the specific contributions of each individual marker component to ZYSD's overall effects remain to be clarified in future studies.

Currently, there are 3 main models for studying the effects of drugs on myocardial infarction. The first involves inducing myocardial infarction with drugs such as isoproterenol. Isoproterenol is a β‐adrenergic receptor agonist commonly used in animal models to induce myocardial infarction. The mechanism of isoproterenol‐induced myocardial infarction mainly involves an increase in oxidative stress, leading to the induction of myocardial infarction and subsequent cellular toxicity. The continuous presence of these oxidative stress factors can further impact the function of myocardial tissue. Free radicals generated as a result initiate lipid peroxidation, causing irreparable damage to the myocardial membrane (Davel et al. 2014). Isoproterenol‐induced myocardial necrosis results in changes in membrane permeability, leading to the loss of structure and function in the membrane and tissues constituting the myocardium. The pathophysiological and structural changes in the rat heart in this model are similar to human myocardial infarction events. The advantage of this model lies in its simple modeling process, which only requires subcutaneous injections of isoproterenol once a day for two consecutive days. The modeling success rate is relatively high. After modeling, there is minimal variation in myocardial infarction and heart function among individuals, providing good model stability. Therefore, isoproterenol is widely used in the study of ischemic myocardial infarction.

A second method involves establishing a model via ligation of the left anterior descending branch of the left ventricle through open‐chest surgery (Wu et al. 2011). The primary advantage of this method is its close resemblance to the clinical pathophysiology of ischemic myocardial infarction caused by occlusion of the left anterior descending coronary. However, our preliminary experiments identified notable disadvantages. The surgical procedure is technically demanding, requiring precise ligation of the coronary artery while the heart remains beating. This process is associated with a high risk of fatal complications, including puncture or transection of the left anterior descending artery by the suture needle. After the animal model was established, due to the difficulty in ensuring consistency in the ligated anterior descending branch area, there was a significant individual variation in the infarct size of the left ventricle and cardiac function, leading to poor model stability and making it unfavorable for the assessment of intervention effects. Post open‐chest surgery, rats were susceptible to death from infection, which affects the analysis of the cause of death, which complicated the analysis of mortality causes.

The third approach is the ex vivo heart model (Kuptsova et al. 2022). In this model, the animal heart is excised and perfused using a Langendorff‐Isolated perfusion system to maintain cardiac activity for a period of time. It allows for pre‐interventions before establishing the perfusion system or interventions during the process when the system is sustaining the heartbeat. The key advantage includes the ease of real‐time data monitoring, and the ability to perform precise ligation of the left coronary anterior descending coronary artery, making it particularly suitable for studying drugs with rapid onset of action. The main limitation is the relatively short duration of viable cardiac function, which restricts the observation of the long‐term efficacy. We aimed to evaluate the effect of ZYSD on ischemic myocardial infarction. Therefore, we required an animal model with stable cardiac pathological changes and cardiac functional damage. This led to the exclusion of the open‐chest ligation method of the left coronary anterior descending branch for this study. Furthermore, considering the potential intervention of food through the gut microbiota on ischemic myocardial infarction, we needed to collect rat feces during the animal study process. Consequently, this study did not employ the Langendorff‐Isolated perfusion system but instead adopts a modeling protocol involving subcutaneous injection of isoproterenol. It should be noted that the choice of model may limit the generalizability of our findings to clinical scenarios.

In the exploration of the preventive effects of ZYSD on isoproterenol‐induced myocardial infarction, the mortality rate in each group of animals was approximately 30%. Due to the relatively small sample size, this experiment could not conclusively determine whether various intervention methods could reduce the mortality rate induced by isoproterenol‐induced myocardial infarction. Most of the deceased animals died after the first injection of isoproterenol, which may be attributed to myocardial infarction and arrhythmias caused by subcutaneous injection of a high dose of isoproterenol. Ultrasound echocardiography was used to assess cardiac function in rats, revealing that medium and high‐dose ZYSD significantly improved heart function, manifested by increased ejection fraction and shortened fractional shortening. The electrocardiogram results also suggested that ZYSD improved characteristic waveforms associated with isoproterenol‐induced P‐wave changes and ST‐segment elevation, typical of myocardial infarction. Further examination of cardiac histopathology using H&E staining demonstrated that ZYSD pretreatment significantly reduced the area of myocardial infarction and decreased inflammatory cells, confirming our predictions based on network pharmacology. Based on these findings, serum levels of CK and CK‐MB, two indicators associated with myocardial infarction, were measured. ZYSD was found to significantly downregulate the levels of CK and CK‐MB in the serum, suggesting that ZYSD pretreatment could reduce isoproterenol‐induced myocardial cell necrosis. In addition, in conjunction with the protein–protein interaction network in network pharmacology, we assessed the levels of inflammation‐related markers TNF‐α, IL‐1β, and IL‐6. ZYSD was found to downregulate the levels of TNF‐α, IL‐1β, and IL‐6 in the myocardium.

Since the mechanism of isoproterenol‐induced myocardial infarction is primarily associated with oxidative stress, and network pharmacology studies also suggested that ZYSD may intervene in myocardial infarction by regulating oxidative stress, we measured oxidative stress‐related indicators such as SOD, GSH, TBARS, and LOOH in the myocardium. SOD and GSH in tissues have antioxidant effects, reducing tissue oxidative toxicity. On the other hand, TBARS and LOOH are marker products indicative of cellular damage due to oxidative stress (Georgiou‐Siafis and Tsiftsoglou 2023). ZYSD up‐regulated SOD and GSH in the myocardium and downregulated TBARS and LOOH in the myocardium. This suggests that ZYSD may alleviate oxidative stress by influencing the expression of antioxidants in the myocardium. In the exploration of post‐intervention with ZYSD in isoproterenol‐induced myocardial infarction experiments, the results obtained were similar to the results of the aforementioned pre‐intervention experiments. This indicates that both pre‐intervention and post‐intervention with ZYSD can improve isoproterenol‐induced ischemic myocardial infarction by modulating oxidative stress and inflammatory responses in myocardial tissue. However, these findings are limited to the isoproterenol‐induced model and may not be applicable to other types of myocardial infarction.

ZYSD was administered via gavage, with its bioactive constituents being absorbed through the intestinal tract. The consumption of edible substances with medicinal attributes may introduce compounds that interact with the gut microbiota. Notably, the interaction between the drug and the microbiota could result in the modulation of specific bacterial genera's relative abundance. Certain bacterial genera were found to metabolize the active ingredients of ZYSD, leading to structural modifications that could potentially alter their pharmacological activities. Furthermore, the protective effect of ZYSD against isoproterenol‐induced myocardial infarction exhibited a concentration‐dependent trend. This variation might be associated with the influence of different concentrations of ZYSD on the bacterial populations capable of interacting with its active components, though alternative mechanisms cannot be excluded.

The major representative components contained in ZYSD, including icariin, epimedin A, epimedin B, epimedin C, liquiritin, and quercetin, belong to the flavonoid category, while glycyrrhetinic acid and ginsenosides belong to the triterpenoid category, and salvianolic acid C belongs to the phenolic acid category. These three categories of substances all possess anti‐inflammatory and antioxidant effects (Chagas et al. 2022; Fu et al. 2018; Chien et al. 2021). We performed a 16SrDNA sequencing on rat cecal contents to screen for bacterial genera associated with oxidative stress or inflammation that can also interact with flavonoid, triterpenoid, or phenolic acid substances. The results demonstrated that the relative abundance of *Lactobacillus*, *Bifidobacterium*, and *Clostridium* in the PostHZ group was significantly up‐regulated. These three types of bacterial genera have a closer interaction with the aforementioned three categories of substances. *Lactobacillus* has an improving effect on inflammation and oxidative stress (Rastogi and Singh 2022). Flavonoid substances, triterpenoid substances such as ginsenosides, and phenolic acid substances can increase the relative abundance of *Lactobacillus* (Rodriguez‐Casta et al. 2019; Chen et al. 2022; Gong et al. 2020). Correspondingly, *Lactobacillus* can enhance the bioavailability of flavonoid substances like quercetin and triterpenoid substances like ginsenosides (Chen et al. 2022; Park et al. 2021). All dosage groups of ZYSD significantly increased the relative abundance of *Lactobacillus* in cecal contents.

The interaction between ZYSD and *Lactobacillus* may be one of the factors in alleviating isoproterenol‐induced ischemic myocardial infarction in rats. Similar to *Lactobacillus*, *Bifidobacterium* can also alleviate inflammation and oxidative stress (Achi et al. 2019; Vitheejongjaroen et al. 2022). Flavonoids and triterpenoids, as well as phenolic acid substances, can increase the relative abundance of *Bifidobacterium* (Chen et al. 2022; Gong et al. 2020; Gwiazdowska et al. 2015). *Bifidobacterium* can transform less effective flavonoid substances into more effective ones. For example, substances with less efficacy such as *Epimedium B* and *Epimedium C* can be transformed into more effective substances like icariin (Su et al. 2023). Additionally, *Bifidobacterium* can promote the transformation of triterpenoids, such as ginsenosides, and enhance the absorption rate of ginsenosides (Chen et al. 2022). In this study, it was observed that the relative abundance of *Bifidobacterium* in the cecal contents of rats with isoproterenol‐induced myocardial infarction significantly decreased. A high dose of ZYSD significantly increased the relative abundance of *Bifidobacterium*, whereas no significant changes were noted in the relative abundance of *Bifidobacterium* in the medium and low dose groups. This indicates that a high dose of ZYSD may facilitate the growth and colonization of *Bifidobacterium*. As mentioned above, *Bifidobacterium* may enhance the efficacy and absorption rate of certain active components in ZYSD. The lack of significant improvement in the relative abundance of *Bifidobacterium* in the medium and low dose groups may be the reason why some key active components fail to be converted into more effective or higher absorption rate substances in the intestine. This could be one of the reasons why the therapeutic effect of ZYSD on ischemic myocardial infarction shows a concentration‐dependent gradient.

It has been found that *Clostridium* can participate in the metabolism of various active components in ZYSD, such as quercetin and glycyrrhizic acid (Rodriguez‐Casta et al. 2019; Keranmu et al. 2022). Intestinal bacteria can express a variety of oxidoreductases, and various flavonoid substances complete the transfer of electrons or hydrogen atoms with the help of these enzymes. The reduction reaction during the metabolic transformation of flavonoid substances in the body can increase their bioavailability and pharmacological activity. For example, the flavonoid reductase in *Clostridium orbiscindens* can catalyze the hydrogenation of the double bonds at the C2 and C3 positions on the C ring structure of flavones and flavonols, generating dihydroflavones and dihydroflavonols (Yang et al. 2021). The reduction reaction that occurs after the deglycosylation of flavonols follows the metabolic rules of compounds such as quercetin; quercetin undergoes degradation under the action of *Clostridium orbiscindens* , reducing the double bond between C2 and C3 of the C ring, and cleaving the oxygen atom to form the corresponding tautomeric compound, chalcone (Feng et al. 2018), which has good anti‐inflammatory and antioxidant effects (Mahapatra et al. 2017; Egbujor et al. 2021). In this study, it was found that the relative abundance of *Clostridium* in the cecal contents increased with the dose of ZYSD. This may be an important reason related to the gut microbiota for the intervention effect of ZYSD on ischemic myocardial infarction showing a concentration‐dependent gradient.

This study has several limitations. ZYSD has a slightly irritating taste, while a pleasant taste sensation is a key characteristic of high‐quality beverages. In subsequent experiments, we will explore methods to improve ZYSD's taste while preserving its therapeutic efficacy. Moreover, due to the complex interactions between the gut microbiota and the active components of ZYSD, we have only selected representative flavonoids, triterpenoids, and phenolic acids from the beverage, as well as *Lactobacillus*, *Bifidobacterium*, and *Clostridium*, which show significant differences in abundance among groups, as examples to briefly discuss the interactions between the gut microbiota and the active components in ZYSD. Consequently, we do not elaborate on the potential interactions between other active components and other bacterial genera.

Utilizing network pharmacology, we predicted the interventional feasibility of ZYSD in ischemic myocardial infarction and identified its potential targets. The analysis revealed that ZYSD components could interact with 96 targets related to ischemic myocardial infarction, with a primary focus on inflammation‐related targets such as TNF, IL‐6, and IL‐1β. Biological process prediction suggested that ZYSD's impact on ischemic myocardial infarction is primarily through modulating oxidative stress and inflammatory damage. These insights were validated through animal experiments, which supported our hypothesis that ZYSD intervenes in ischemic myocardial infarction by affecting oxidative stress and inflammatory damage.

The expression of TNF‐α, IL‐1β, and IL‐6 is closely linked to the activation of the TLR4/NF‐κB pathway, which subsequently facilitates the translocation of NF‐κB to the nucleus (Kawai and Akira 2010). Therefore, we assessed the expression levels of the TLR4/NF‐κB pathway in the myocardium. In this study, we evaluated the myocardial expression levels of key components within the TLR4/NF‐κB pathway. Our findings suggest that ZYSD potentially exerted its therapeutic effect by suppressing the myocardial levels of TNF‐α, IL‐1β, and IL‐6 through the inhibition of the TLR4/NF‐κB pathway. This modulation of the pathway represented a critical mechanism by which ZYSD exerts its cardioprotective properties. The expression of SOD and GSH is associated with the activation of Nrf2 (Li et al. 2016). Nrf2 plays an essential role in the detection of oxidative stress and the regulation of antioxidant responses, which is modulated by the PI3K/Akt signaling pathway (Yin et al. 2015). Our investigational data propose that ZYSD may mitigate isoproterenol‐induced oxidative stress in the myocardium by enhancing the expression of SOD and GSH. This up‐regulation is hypothesized to occur through the activation of the PI3K/Akt/Nrf2 signaling axis, thereby bolstering the cellular antioxidant defenses against oxidative damage. Moreover, molecular docking analysis in our study showed that some of the active ingredients identified by UPLC bind well to the core targets AKT and NRF2, which further verified that ZYSD exerts a good regulatory effect on the PI3K/Akt/Nrf2 signaling axis. Similarly, it is reported that ginsenoside Rb3 simultaneously promotes both JNK‐mediated NF‐κB and PERK/Nrf2/HMOX1 regulation of oxidative stress, inflammation, and cell apoptosis to improve myocardial infarction (Ramli et al. 2022). Besides, other active ingredients identified in the HPLC of ZYSD including liquiritin, salvianolic acid C, glycyrrhetinic acid and so on, have a protective effect on ischemic tissue damage (Chu et al. 2021; Han et al. 2022; Yang et al. 2022), indicating the potential mechanism by which ZYSD exerts its effects is through these active ingredients.

Metoprolol primarily reduces inflammation and oxidative stress damage associated with ischemic myocardial infarction through its negative inotropic effects, while ZYSD may alleviate such damage by supplying the body with anti‐inflammatory and antioxidant components following its interaction with the gut microbiota. The combined use of these 2 agents may exert a synergistic effect, and we will further confirm this hypothesis in future clinical trials.

## 5 Conclusion

This study found that both pre‐intervention and post‐intervention with ZYSD can improve the reduced cardiac function and electrocardiogram abnormalities induced by isoproterenol‐induced ischemic myocardial infarction, exhibiting a dose‐effect relationship positively correlated with its concentration. The effects of medium and high doses of ZYSD are similar to those of the positive control drug, metoprolol. Based on network pharmacology studies, we found that ZYSD primarily ameliorates ischemic myocardial infarction by targeting pathways related to inflammation and oxidative stress. The intervention effect of ZYSD on ischemic myocardial infarction exhibits a dose‐effect relationship positively correlated with its concentration, which may be associated with the interactions between ZYSD and the gut microbiota, specifically *Lactobacillus*, *Bifidobacterium*, and *Clostridium*. As ZYSD is composed of food‐derived ingredients, it can provide an effective and safer alternative for the prevention or treatment of ischemic myocardial infarction.

## Author Contributions

**Kechao Nie:** investigation, writing – original draft. **Donghua Liu:** investigation, writing – original draft. **Zhuotao Fu:** supervision, conceptualization, writing – original draft, writing – review and editing. **Linchun Fu:** supervision, writing – original draft. **Tian Yu:** investigation, writing – original draft. **Jiawen Huang:** investigation, data curation, writing – original draft, writing – review and editing. **Guoyi Li:** investigation, writing – original draft. **Cong Meng:** formal analysis, writing – original draft. **Zhitong Deng:** conceptualization, investigation, supervision, writing – review and editing, writing – original draft. **Liang Long:** methodology, visualization, software, writing – original draft. **Xiaoling Shen:** supervision, writing – original draft. **Yingjie Hu:** conceptualization, supervision, writing – review and editing, writing – original draft.

## Funding

This work was financially supported by the Projects of Guangzhou University of Chinese Medicine (grant number ‐ GZYFH2025G01 to Y.H.), National Key Research and Development Plan Project of China (grant number 2019YFE0109800 to Y.H.), Guangzhou Municipal Health Technology Project (grant number 20222A011005 to C.M.), and project supported by Hainan Province Clinical Medical Center (no grant number to Z.D.).

## Conflicts of Interest

The authors declare no conflicts of interest.

## Supporting information


**Supplementary Figure 1.** The image of ZYSD.
Supplementary Figure 2. ZYSD and metoprolol can both improve the electrocardiographic manifestations of acute myocardial infarction.
Supplementary Figure 3. ZYSD and metoprolol can both improve the electrocardiographic manifestations of acute myocardial infarction.
Supplementary Table 1. The origins and lot numbers of the TCM decoction pieces.
Supplementary Table 2. Mobile Phase Elution Gradient.
Supplementary Table 3. The Retention time for reference compounds.
Supplementary Table 4. The mortality rate of isoproterenol‐induced myocardial infarction in rats.
Supplementary Table 5. The main components and targets of ZYSD in alleviating ischemic myocardial infarction.
Supplementary Table 6. The binding free energy (in kcal/mol) and its components obtained from the MM/GBSA calculation for AKT1‐Epimedin C, AKT1‐Icariin, AKT1‐Salvianolic acid c and AKT1‐Quercitrin complex model.

## Data Availability

The datasets used and/or analyzed during the current study are available from the corresponding author on reasonable request.
