# Supplementary material for: Zhenyuan Solid Drink Ameliorates Inflammation and Oxidative Stress in Isoproterenol‐Induced Ischemic Myocardial Infarction via TLR4/NF‐κB Pathway and PI3K/AKT1/NRF2 Pathway in Rats
Source: Food Sci Nutr. 2026 Apr 19;14(4):e71798. doi: 10.1002/fsn3.71798 (PMC13092805; doi:10.1002/fsn3.71798)
Supplement: Supplementary file 1 — Supplementary Figure 1. The image of ZYSD. Supplementary Figure 2. ZYSD and metoprolol can both improve the electrocardiographic manifestations of acute myocardial infarction. Supplementary Figure 3. ZYSD and metoprolol can both improve the electrocardiographic manifestations of acute myocardial infarction. Supplementary Table 1. The origins and lot numbers of the TCM decoction pieces. Supplementary Table 2. Mobile Phase Elution Gradient. Supplementary Table 3. The Retention time for reference compounds. Supplementary Table 4. The mortality rate of isoproterenol‐induced myocardial infarction in rats. Supplementary Table 5. The main components and targets of ZYSD in alleviating ischemic myocardial infarction. Supplementary Table 6. The binding free energy (in kcal/mol) and its components obtained from the MM/GBSA calculation for AKT1‐Epimedin C, AKT1‐Icariin, AKT1‐Salvianolic acid c and AKT1‐Quercitrin complex model. [file FSN3-14-e71798-s001.docx]

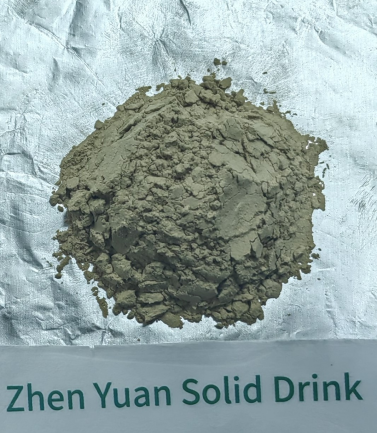


**Supplementary Fig.1.** The image of ZYSD.


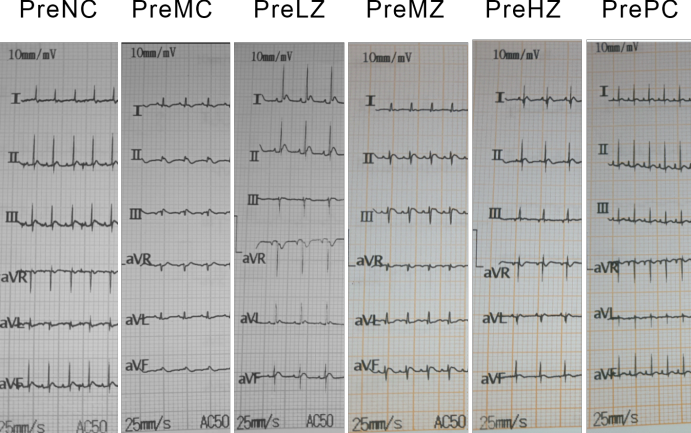


**Supplementary Fig.2.** ZYSD and metoprolol can both improve the electrocardiographic manifestations of acute myocardial infarction.


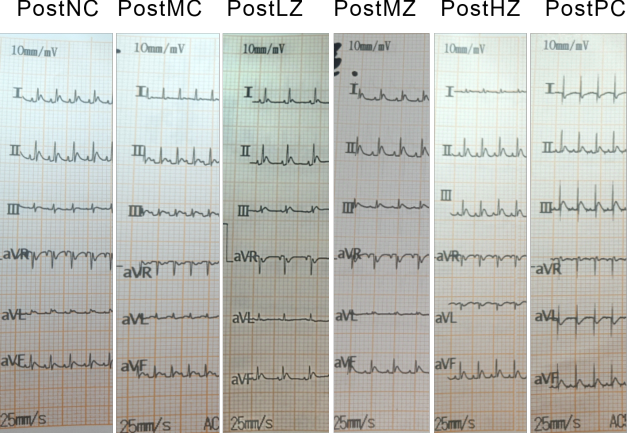


**Supplementary Fig.3.** ZYSD and metoprolol can both improve the electrocardiographic manifestations of acute myocardial infarction.

**Supplementary Table 1. The origins and lot numbers of the TCM decoction pieces.**

| Serial number | Name of product | Place of origin | Lot number |
| --- | --- | --- | --- |
| 1 | *Epimedium* | Gansu (Province) | 211201 |
| 2 | *Zingiberis Rhizoma* | Yunnan (Province) | 211001 |
| 3 | *Glycyrrhiza* | Gansu (Province) | 211001 |
| 4 | *Panax Ginseng* | Jilin (Province) | 210901 |
| 5 | *S. Miltiorrhiza* | Hebei (Province) | 211201 |
| 6 | *Poria Cocos* | Yunnan (Province) | 210801 |

**Supplementary Table 2. Mobile Phase Elution Gradient.**

| Time/min | Mobile Phase A（%） | Mobile Phase B(%) |
| --- | --- | --- |
| 0-10 | 10→22 | 90→78 |
| 10-25 | 22→46 | 78→54 |
| 25-35 | 46→90 | 54→10 |
| 35-40 | 90→90 | 10→10 |
| 40-41 | 90→10 | 10→90 |
| 41-42 | 10→10 | 90→90 |

**Supplementary Table 3. The Retention time for reference compounds.**

| Time/min | Mobile Phase A（%） | Mobile Phase B(%) |
| --- | --- | --- |
| 1 | Liquiritin | 9.43 |
| 2 | Ginsenosides Rb3 | 15.47 |
| 3 | Epimedin A | 19.81 |
| 4 | Epimedin B | 20.07 |
| 5 | Salvianolic acid C | 20.27 |
| 6 | Epimedin C | 20.69 |
| 7 | Icariin | 20.99 |
| 8 | Quercetin | 21.83 |
| 9 | Glycyrrhetinic acid | 25.97 |
| 10 | Tanshinone IIA | 35.93 |

**Supplementary Table 4. The mortality rate of isoproterenol-induced myocardial infarction in rats.**

|  | | | Group | | | | | | Total |
| --- | --- | --- | --- | --- | --- | --- | --- | --- | --- |
|  |  |  | PreNC | PreMC | PreLZ | PreMZ | PreHZ | PrePC |  |
| Condition | Alive | Count | 6 | 6 | 5 | 5 | 6 | 6 | 34 |
|  |  | % within group | 100% | 66.7% | 71.4% | 71.4% | 75% | 75% | 75.6% |
|  | Die | Count | 0 | 3 | 2 | 2 | 2 | 2 | 11 |
|  |  | % within group | 0% | 33.3% | 28.6% | 28.6% | 25% | 25% | 24.4% |
| Total |  | Count | 6 | 9 | 7 | 7 | 8 | 8 | 45 |
|  |  | % within group | 100% | 100% | 100% | 100% | 100% | 100% | 100% |

7 cells (58.3%) have expected count less than 5. The minimum expected count is 1.47.

The standardized statistic is 0.536

Fisher’s Exact Test: P=0.334, value=2.736

**Supplementary Table 5. The main components and targets of ZYSD in alleviating ischemic myocardial infarction.**

| Serial number | Ingredients | Number of targets | Source of traditional Chinese medicine  (latin name) |
| --- | --- | --- | --- |
| 1 | Quercetin | 69 | *Glycyrrhiza*, *Epimedium* |
| 2 | Kaempferol | 26 | *Glycyrrhiza*, *Panax Ginseng*, *Epimedium* |
| 3 | Luteolin | 23 | *Salvia Miltiorrhiza*, *Epimedium* |
| 4 | Naringenin | 16 | *Glycyrrhiza, Poria Cocos* |
| 5 | 7-Methoxy-2-methyl isoflavone | 15 | *Glycyrrhiza* |
| 6 | β-sitosterol | 15 | *Glycyrrhiza, Zingiberis Rhizoma, Poria Cocos, Epimedium* |
| 7 | Anhydroicaritin | 14 | *Epimedium* |
| 8 | Isorhamnetin | 14 | Glycyrrhiza |
| 9 | 8-(3-methylbut-2-enyl)-2-phenyl-chromone | 14 | *Epimedium* |
| 10 | Tanshinone iia | 13 | *S. Miltiorrhiza* |

**Supplementary Table 6. The binding free energy (in kcal/mol) and its components obtained from the MM/GBSA calculation for AKT1-Epimedin C, AKT1-Icariin, AKT1-Salvianolic acid c and AKT1-Quercitrin complex model.**

| Type | AKT1-Epimedin C | AKT1-Icariin | AKT1-Salvianolic acid c | AKT1-Quercitrin |
| --- | --- | --- | --- | --- |
| Δ*E_vdw_* | -36.7103±0.3753 | -34.9704±0.4082 | -25.8852±0.3972 | -34.0714±0.2938 |
| Δ*E_ele_* | -98.3221±0.9146 | -87.5719±1.2190 | -93.9889±1.1255 | -68.6051±0.8687 |
| Δ*G_polar_* | 99.7203±0.5613 | 90.4170±0.6830 | 92.5556±0.8336 | 83.8568±0.8138 |
| Δ*G_nonpolar_* | -7.3297±0.0266 | -6.9895±0.0235 | -5.4441±0.0246 | -5.3386±0.0180 |
| Δ*G_binding_* | -42.6418±0.5237 | -39.1148±0.4952 | -32.7626±0.3299 | -24.1582±0.2988 |

Note: The contribution to the binding free energy (ΔG_total_) from the Van der Waals and electrostatic interactions is represented byΔE_vdw_ andΔE_ele_, respectively. The polar and nonpolar solvation energy contributions toΔG_binding_ are represented byΔG_polar_ andΔG_nonpolar_, respectively.
